# Root Endophytes and *Ginkgo biloba* Are Likely to Share and Compensate Secondary Metabolic Processes, and Potentially Exchange Genetic Information by LTR-RTs

**DOI:** 10.3389/fpls.2021.704985

**Published:** 2021-07-09

**Authors:** Kai Zou, Xueduan Liu, Qi Hu, Du Zhang, Shaodong Fu, Shuangfei Zhang, Haonan Huang, Fangying Lei, Guoqing Zhang, Bo Miao, Delong Meng, Luhua Jiang, Hongwei Liu, Huaqun Yin, Yili Liang

**Affiliations:** ^1^School of Minerals Processing and Bioengineering, Central South University, Changsha, China; ^2^Key Laboratory of Biometallurgy of Ministry of Education, Changsha, China; ^3^NEOMICS Institute, Shenzhen, China; ^4^Shenzhen Agricultural Genome Research Institute, Chinese Academy of Agricultural Sciences, Shenzhen, China

**Keywords:** *Ginkgo biloba*, endophyte, secondary metabolism, LTR-RT, lateral gene transfer

## Abstract

*Ginkgo biloba* is a pharmaceutical resource for terpenes and flavonoids. However, few insights discussed endophytes’ role in *Ginkgo*, and whether genetic exchange happens between *Ginkgo* and endophytes remains unclear. Herein, functional gene profiles and repetitive sequences were analyzed to focus on these issues. A total of 25 endophyte strains were isolated from the *Ginkgo* root and distributed in 16 genera of 6 phyla. Significant morphological diversities lead to the diversity in the COG functional classification. KEGG mapping revealed that endophytic bacteria and fungi potentially synthesize chalcone, while endophytic fungi might also promote flavonoid derivatization. Both bacteria and fungi may facilitate the lignin synthesis. *Aspergillus* sp. Gbtc_1 exhibited the feasibility of regulating alcohols to lignans. Although *Ginkgo* and the endophytes have not observed the critical levopimaradiene synthase in ginkgolides synthesis, the upstream pathways of terpenoid precursors are likely intact. The MVK genes in *Ginkgo* may have alternative non-homologous copies or be compensated by endophytes in long-term symbiosis. *Cellulomonas* sp. Gbtc_1 became the only bacteria to harbor both MEP and MVA pathways. Endophytes may perform the mutual transformation of IPP and DMAPP in the root. *Ginkgo* and bacteria may lead to the synthesis and derivatization of the carotenoid pathway. The isoquinoline alkaloid biosynthesis seemed lost in the *Ginkgo* root community, but L-dopa is more probably converted into dopamine as an essential signal-transduction substance. So, endophytes may participate in the secondary metabolism of the *Ginkgo* in a shared or complementary manner. Moreover, a few endophytic sequences predicted as *Ty3/Gypsy* and *Ty1/Copia* superfamilies exhibited extremely high similarity to those of *Ginkgo*. CDSs in such endophytic LTR-RT sequences were also highly homologous to one *Ginkgo* CDS. Therefore, LTR-RTs may be a rare unit flowing between the *Ginkgo* host and endophytes to exchange genetic information. Collectively, this research effectively expanded the insight on the symbiotic relationship between the *Ginkgo* host and the endophytes in the root.

## Introduction

Ever since discovering and separating microorganisms, humanity has opened a new world of cognition and scientific research. Microorganisms can be distributed on the seabed (Morono et al., 2020), atmosphere (Lighthart, 2006), deep soil (Zheng et al., 2017), and even survive in other extreme environments, such as the Antarctic ice (Lanoil et al., 2009) and volcanic craters (Wahyuntari et al., 2000). Notably, some are defined as endophytes because they almost exist in all animals and plants. With the unique physiological characteristics, such as long lifespan and stable environmental tolerance, higher plants have been chosen to extensively study the multidimensional interactions between the endophytes and their hosts.

It is commonly accepted that the existing plants (∼300,000 species) each hosts up to several hundred species of endophytes (Strobel and Daisy, 2003). In terms of life strategies, these endophytes can be classified into obligate and facultative ones (Hardoim et al., 2008). The obligate endophytes strictly rely on the plant host to grow and survive, and they are transmitted vertically or by carriers. For example, *Xylella fastidiosa* exhibits well adaption to the life in plant xylem, but can cause disease after transmitted to *Citrus sinensis* L. via insect vectors (Chang et al., 1993; Araujo et al., 2002). However, facultative endophytes refer to the ones that enter the plant at a particular life stage and then gradually survive and persist in the host. The rhizobia are the representative facultative endophytes, and they are considered to be derived from soil and invade plant cells or tissues through cracks at the fibrous root junction (Chi et al., 2005). Root crack may be the primary approach for endophytes colonization; notwithstanding, other entries into the plant host still exist, like stomata on leaf tissue and physical trauma (McCully, 2001).

In general, endophytes’ effects on plants mainly include several aspects. Firstly, some microbes may synthesize plant hormones, such as indole-3-acetic acid (IAA), gibberellins (Gas) and cytokinins (CKs), to promote growth (Glick, 2012), and also be able to modulate the ethylene levels by degrading 1-aminocyclopropane-1-carboxylate (ACC) or inhibiting ACC synthase in the life stage (Sugawara et al., 2006; Glick et al., 2007). Secondly, like their host, endophytes can produce common secondary metabolites of bioactivity, i.e., antibiotics (Martinez-Klimova et al., 2017), anticancer camptothecin (Puri et al., 2005), and podophyllotoxin (Puri et al., 2006). Also, a fraction of them may activate and regulate their host’s secondary metabolism; for instance, the endophytes *Acinetobacter* sp. and *Marmoricola* sp. became the confirmed parasite to upregulate critical genes in benzylisoquinoline alkaloid biosynthesis in *Papaver somniferum* L. Besides, eliciting the host response and secreting anti-phytopathogen substances to enhance biotic-stresses resistance could be another benefit of endophytes for plant hosts. *Fusarium solani* was reported to stimulate induced systemic resistance (ISR) to resist pathogen *Septoria lycopersici* by inducing pathogenesis-related gene expression in tomato root tissues (Kavroulakis et al., 2007). Such as *Pseudomonas putida* BP25 (Sheoran et al., 2015) and *Rhizobium meliloti* (Arora et al., 2001), some endophytic bacteria can produce various volatile compounds with antimicrobial activity against phytopathogenic bacteria, fungi, and nematodes. Moreover, plant endophytes can induce and intensify the resistance to abiotic stress. Endophytic bacteria reduces metal phytotoxicity via extracellular precipitation, intracellular accumulation, sequestration, or biotransformation of toxic metal ions to less toxic or non-toxic forms (Khare et al., 2018). Some fungal species can help the plants adapt to abiotic stress by increasing resistance to drought or water stress, high temperature, and high salinity (Redman et al., 2002; Waller et al., 2005; Bae et al., 2009). Therefore, endophytes are an eco-friendly choice that promotes plant growth and serves as a sustainable resource for bioactive natural products.

*Ginkgo biloba* (*Ginkgo*) is an ancient living plant with a unique advantage in scientific research. Its abundant pharmacological components, terpenoids and flavonoids, lead to an excellent inhibitory effect on many pathogenic microorganisms (Lee et al., 2014). Apart from several strains belonging to *Nocardioides ginkgobilobae* (Xu et al., 2016), *Fusarium oxysporum* (Cui et al., 2012) and *Penicillium* sp. (Yuan et al., 2014), most *Ginkgo* endophytes were isolated from the leaves, branches, or seeds rather than the roots, for example, *Streptomyces* and *Pseudochaetosphaeronema* species (Zhang et al., 2016; Yan et al., 2018).

Quite a few studies have discussed the metabolites of *Ginkgo* endophytes, including many biologically active ingredients, such as flavonoids (Qiu et al., 2010; Yan et al., 2013), terpenoids (Cui et al., 2012; Yan et al., 2013; Qian et al., 2016) and alkaloids (Zhang et al., 2013), as well as a series of volatile organic compounds (VOCs), like butanol and acetate (Banerjee et al., 2010). In addition, chaetoglobosins (Li et al., 2014), chaetomugilin (Qin et al., 2009a), altertoxin (Qin et al., 2009b), etc., were also discovered in *Ginkgo* endophytes as cytotoxic ingredients. These metabolites have made outstanding contributions to *Ginkgo*’s resistance to abiotic stress and resistance to pests and diseases.

So, many studies have been carried out on the metabolites of *Ginkgo* endophytes, but few insights were discussed on the detailed mechanism of how endophytes function in the symbiosis with *Ginkgo*, especially in the flavonoids and terpenoids biosynthesis processes. It is reported that the flavonoid accumulation in suspension cells of *Ginkgo* can be induced by the abscisic acid (ABA) from fungal endophytes (Hao et al., 2010), which pushed us not to ignore the potential effect of endophytes in the synthesis and regulation process of flavonoids. On the other hand, symbiosis and genetic evolution are closely linked with some signs that indicated the feasibility of genetic exchange between the host and parasites (Hotopp et al., 2007; Moran and Jarvik, 2010). *Arabidopsis thaliana* is reported to harbor several GTPase sequences ingested from cyanobacteria and α-proteobacteria through the endosymbiotic gene transfer (EGT) process (Suwastika et al., 2014), which is one of the scarce genetic exchange authentications between plants and endophytes. To date, no relevant studies have uncovered the evolutionary genetic relationship between the world-famous *Ginkgo* species and endophytes. Therefore, in this study, wild *Ginkgo* roots were sampled to isolate bacterial and fungal endophytes. Based on their draft genomes, bioinformatics analyses were achieved to explore the potential function of endophytes in the secondary metabolic pathways of *Ginkgo* roots and probe into the possible genetic exchanges between them. We hope this work can provide a novel perspective on the relationship between plants and microorganisms.

## Materials and Methods

### Ginkgo Tree Choice and Root Tissues Collection

The *Ginkgo* trees grow wildly in Linyi City, Shandong Province, China. The detailed location is 34°36′34′′ N, 118°12′8′′ E with an altitude of 40 m. Generally, the diameter at breast height (DBH) represents the relative extent of tree ages in the same (or similar) environment (Zou et al., 2019) according to the formula (age of tree = diameter ^∗^ growth factor) proposed by the International Society of Arboriculture^1^. The presumptive oldest healthy *Ginkgo* tree with the largest DBH (0.64 m) in this area was chosen to collect the root materials.

**FIGURE 1:**
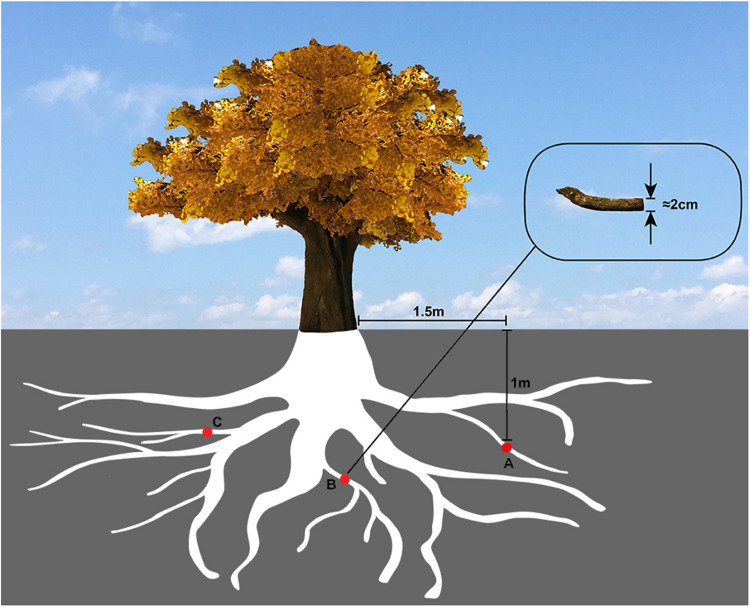
The sampling diagram of *Ginkgo* roots. The red circles **(A,B,C)** represented the *Ginkgo* roots’ sampling sites, which were evenly located around the tree, 1.5 m horizontally away from the trunk and 1 m deep underground.

Evenly located around the tree, a total of three locations were selected to collect the samples. Several root tissues of 2 cm diameter were cut off from the part of the tree, which was 1.5 m horizontally away for the trunk and 1m deep underground (Figure 1), and then mixed to make a composite sample.

### Endophytes Isolation

The rhizosphere soil of the sampled roots was removed by physical treatment, including hand shaking, hairbrush cleaning, and thoroughly water washing. Then, successively, all tissues were rinsed in sterile distilled water for six times, 70% ethanol for 2 min, 5.25% sodium hypochlorite for 4 min, and sterile distilled water for five times at last (Rustamova et al., 2020). In order to confirm the complete sterilization of the surface, 100 μl of the final eluate is reserved for plate culture as the control.

Afterward, these preprocessed root tissues were physically fragmented mildly in the phosphate buffer saline (PBS), which was used to protect and suspend endophytic microorganisms during the physical fragmentation (Liao and Shollenberger, 2003; Hoang et al., 2020). At last, the obtained cell suspension and the final eluate preceding were incubated on selective mediums (initial concentration and 1/10 concentration): (M1) Czapek–Dox Medium (Abildgren et al., 1987), (M2) Potato Dextrose Agar Medium (Phae et al., 1990), (M3) R2A Agar (van der Linde et al., 1999), (M4) 2216E Medium (Patrick, 1978), (M5) Soluble Starch Medium (Jin et al., 2003), (M6) Gauze’s agar medium (El-Nakeeb and Lechevalier, 1963), (M7) LB medium (Baev et al., 2006), (M8) Beef-extract peptone AGAR medium (Kim et al., 2006).

All potentially different colonies are transferred to separate plates with a sterile needle for further culture and purification. Incubated on the same medium as the first time cultivated, all colonies were streak for consecutive generations until no different traits appeared. Then, under a microscope, all these uncontaminated colonies were preliminarily divided into bacteria and fungi according to the colony morphology.

### Endophytes Identification and Filtration

These uncontaminated endophyte strains were respectively collected for DNA extraction using the CTAB method as recommended (Stewart and Via, 1993), among which grinding in the liquid nitrogen is equipped to improve extraction rate and completeness on fungi (Cenis, 1992). The agarose gel electrophoresis and NanoDrop optical density (OD) value calculation were used to assess the quality (purity, concentration, and completeness) of all DNA samples. The pre-distinguished bacteria and fungi were separately carried out the polymerase chain reaction (PCR) with the 16s rDNA and internal transcribed spacer (ITS) primers. The primer sequences were 27F (5′-AGAGTT TGATCCTGGCTCAG-3′) and 1492R (5′-TACGGYTACCTTGTTACGACTT-3′) for 16s rDNA, and ITS1 (5′-TCCGTAGGTGAACCTGCGG-3′) and ITS4 (5′-TCCTCCGCTTATTGATATGC-3′) for ITS. The strains suspected to the same species were selectively abandoned according to the colony morphology coupled with 16s rDNA and ITS blast results. After that, the filtered strains were used for the subsequent genome sequencing and bioinformatics analysis.

### Genome Sequencing, Assembly, and Annotation

The genomic library (average insert size 200–400 bp) was constructed at BGI (Shenzhen, China) and sequenced on the BGISEQ-500 sequencing platform. For enough sequencing depth for assembly, approximately 1 and 5 Gb data were produced severally for bacteria and fungi. To obtain more accurate genomes, the fungal DNA was also used to accomplish sequencing on the PacBio Sequel System at BGI (Shenzhen, China). Then, clean data were yield by removing adapter, ploy-N, and low-quality reads from raw data. The bacterial clean reads were assembled in SPAdes (v3.14.0) (Bankevich et al., 2012) with the default parameters. The fungal clean reads (produced in Pacbio) were assembled in Canu (v1.9) (Koren et al., 2017) to form the draft genomes, which were then polished in NextPolish (v1.0.5) (Hu et al., 2020) based on these PE150 clean reads to be the final genome sequences. CheckM (v1.0.13) (Parks et al., 2015) and BUSCO (v4.1.2) (Simao et al., 2015) were selected to assess the quality of the bacterial and fungal genomes, respectively. Prokka (v1.14.6) (Seemann, 2014) was utilized to achieve gene prediction for bacterial draft genomes, while GeneMark-ES (v4.48_3.60_lic) (Ter-Hovhannisyan et al., 2008) was employed for the fungi. All amino acid sequences were searched in the NCBI non-redundant database (Sayers et al., 2012) in diamond (v2.0.4) (Buchfink et al., 2015). KAAS^2^ (Moriya et al., 2007) was operated for the KEGG annotations with 1E-6 *E*-value. Then, the pathway was then reconstructed in the iPath3 (Darzi et al., 2018). The Clusters of Orthologous Groups of proteins (COG) were predicted in the eggnog-mapper (Huerta-Cepas et al., 2017, 2019).

### Species Classification and Phylogenetic Analysis

Given that 16s rRNA genes (or ITS sequences) were not always enough in species classification in some species of high sequence similarities (Kalia et al., 2016), a composition vector approach without sequence alignment was operated to construct the phylogenetic tree based on the whole genome in CVTree3 (Zuo and Hao, 2015). The K value was set to 6 as recommended (Li et al., 2010). Meanwhile, other 255 species from the GeneBank database were selected for a referential comparison. Finally, the phylogenetic tree was readjusted in MEGA-X (Kumar et al., 2018).

### *Ginkgo* Data Acquisition

As one of the most ancient plants globally, the woody plant *Ginkgo biloba* was predicted to possess a large genome of more than 10 Gb (Zonneveld, 2012). It may be the main reason why its high-quality whole genome was delayed for so many years until it was first released in November 2016 on the *GigaScience* GigaDB repository (Guan et al., 2016) with 41309 CDSs. This study directly downloaded this draft genome with its annotation and repeated sequence information for the following correlation analysis.

### Repetitive Sequences Annotation

Repetitive sequences were elucidated ubiquity in eukaryotes, and its analysis toward all species, including prokaryotes in a specific environment, may help explore possible co-evolution in this community. Here, the repeat library was constructed based on all endophytes’ gathering using the *de novo* prediction program RepeatModeler (V2.0.1) coupled with LtrHarvest (V1.5.9), Ltr_retriever (V2.9.0), MAFFT (V7.471), CD-HIT (V4.8.1), and Ninja (V0.95). Further, the TEclass (Abrusan et al., 2009) platform classified unknown transposable element (TE) consensus sequences into four categories according to their transposition mechanism: DNA transposons, LTRs, LINEs, SINEs. Finally, all classified repeat content was merged as the reference repeat library to annotate all endophytes’ genomes in RepeatMasker (V4.1.1).

### LTR-RT Elements Analysis

The reverse transcriptase domains of *Ty3/Gypsy* and *Ty1/Copia* with sequences EAYLDDLASRSRKRKDHPT HLRLIFERCRYFRIRLNPNKCSFCVTSGRLLGFIVSTTGIMVDP LKVGAIVQLPPPRTIVQLQSLQGKANFLRRFIANYAE and WKVYQMDVKSAFLNGYLEEEVYVQQPPRYEVRGQEDKVY RLKKALNGLKQAPRAWYSKIDSYMIKNEFIRSTSEPTLYTKV NEQGQILIVCLYVDDLIY were used to search all predicted long terminal repeat retrotransposons (LTR-RTs) in the endophytes and the *Ginkgo* host by BLAST (Guan et al., 2016). The resultant sequences annotated as these two LTR-RT superfamilies were extracted to align in Muscle (Edgar, 2004) (V3.8.31) and then construct phylogenetic trees in FastTree (Price et al., 2010) (V2.1.11). Then, the predicted CDSs of LTRs in all isolates were implemented BLASTN against the *Ginkgo* CDSs to obtain the appraised parameters of their similarity.

## Results

### Endophyte Isolation

In total, 63 endophyte strains were isolated from *Ginkgo* root tissues, as listed in Supplementary Table 1. Then, after the suspected same species were selectively abandoned according to the colonies’ morphology (Supplementary Figure 1) and the blast results of the 16s rDNA and ITS sequences (Supplementary File 1), 23 bacterial strains and 2 fungal strains were chosen to be used for the subsequent genome sequencing and bioinformatics analysis. The phylum Actinobacteria included seven strains of four genera, while Firmicutes included nine strains of four genera; both occupied the most of all classified isolates in terms of quantity (Table 1). Of note, the Phylum Proteobacteria screened five genera, which may contain the most considerable phylogenetic diversity.

**TABLE 1:** Species classification and draft genomes details.

ID	Clean Reads	Clean Base	Q20(%)	Scaffolds	N50	Total base	GC (%)	Phylum	Genus	Strain Name	Completeness**	Contamination**
B1	8,077,780	1,211,667,000	91.96	788	58,194	4,464,241	75.09	Actinobacteria	*Cellulomonas*	*Cellulomonas* sp. Gbtc 1	99.42	3.10
B2	8,177,186	1,226,577,900	92.35	2854	217,091	4,364,846	64.91	Actinobacteria	*Microbacterium*	*Microbacterium* sp. Gbtc 1	99.49	4.04
B3	7,780,042	1,167,006,300	92.77	1328	261,072	3,203,135	67.79	Actinobacteria	*Microbacterium*	*Microbacterium* sp. Gbtc 2	98.98	1.04
B4	7,700,302	1,155,045,300	91.20	872	335,249	3,699,314	69.66	Actinobacteria	*Microbacterium*	*Microbacterium* sp. Gbtc 3	99.49	2.71
B5	6,419,050	962,857,500	92.65	683	83,970	2,604,457	72.86	Actinobacteria	*Micrococcus*	*Micrococcus* sp. Gbtc 1	98.70	2.17
B6	8,113,908	1,217,086,200	93.74	603	212,700	12,471,597	70.79	Actinobacteria	*Streptomyces*	*Streptomyces* sp. Gbtc 1	100.00	0.93
B7	7,856,820	1,178,523,000	91.79	846	229,965	12,366,605	70.06	Actinobacteria	*Streptomyces*	*Streptomyces* sp. Gbtc 2	100.00	2.07
B8	8,143,570	1,221,535,500	94.99	570	140,669	8,188,504	47.44	Bacteroidetes	*Chitinophaga*	*Chitinophaga* sp. Gbtc 1	100.00	1.40
B9	6,479,862	971,979,300	94.21	645	176,100	4,432,220	68.85	Deinococcus-Thermus	*Deinococcus*	*Deinococcus* sp. Gbtc 1	100.00	1.13
B10	8,530,228	1,279,534,200	97.40	544	936,783	3,904,465	41.12	Firmicutes	*Bacillus*	*Bacillus* sp. Gbtc 1	100.00	0.98
B11	8,542,264	1,281,339,600	97.29	1026	297,657	5,888,847	35.01	Firmicutes	*Bacillus*	*Bacillus* sp. Gbtc 2	99.18	0.61
B12	8,065,252	1,209,787,800	96.55	1303	360,451	5,633,953	38.15	Firmicutes	*Bacillus*	*Bacillus* sp. Gbtc 3	99.43	5.92
B13	8,075,842	1,211,376,300	94.57	669	2,312,843	4,500,332	45.78	Firmicutes	*Bacillus*	*Bacillus* sp. Gbtc 4	99.59	0.69
B14	8,577,766	1,286,664,900	97.25	370	603,252	5,818,852	35.33	Firmicutes	*Bacillus*	*Bacillus* sp. Gbtc 5	99.00	1.95
B15	7,934,722	1,190,208,300	97.37	647	1,012,143	4,074,767	46.04	Firmicutes	*Bacillus*	*Bacillus* sp. Gbtc 6	99.79	3.89
B16	8,563,006	1,284,450,900	97.55	1046	606,321	4,944,037	37.65	Firmicutes	*Lysinibacillus*	*Lysinibacillus* sp. Gbtc 1	99.34	1.16
B17	8,346,556	1,251,983,400	94.52	608	105,487	8,054,014	58.94	Firmicutes	*Cohnella*	*Cohnella* sp. Gbtc 1	99.18	1.35
B18	8,132,312	1,219,846,800	94.51	1083	450,483	6,778,071	51.84	Firmicutes	*Paenibacillus*	*Paenibacillus* sp. Gbtc 1	99.73	1.56
B19	7,764,668	1,164,700,200	92.78	724	267,255	6,732,356	63.55	Proteobacteria	*Mesorhizobium*	*Mesorhizobium* sp. Gbtc 1	99.51	1.76
B20	5,063,008	759,451,200	95.10	1010	207,593	6,334,492	65.56	Proteobacteria	*Achromobacter*	*Achromobacter* sp. Gbtc 1	99.53	4.27
B21	8,358,042	1,253,706,300	94.32	607	329,603	8,180,439	66.23	Proteobacteria	*Burkholderia*	*Burkholderia* sp. Gbtc 1	100.00	0.80
B22	37,292,636	5,593,895,400	96.37	1316	606,720	5,720,401	54.41	Proteobacteria	*Pantoea*	*Pantoea* sp. Gbtc 1	99.84	2.15
B23	5,589,226	838,383,900	94.43	805	82,214	4,221,366	66.73	Proteobacteria	*Stenotrophomonas*	*Stenotrophomonas* sp. Gbtc 1	99.89	0.90
F1*	33,295,803	4,799,334,750	93.91	18	3,752,118	29,114,197	49.16	Ascomycota	*Aspergillus*	*Aspergillus* sp. Gbtc 1	–	–
F2*	34,022,048	5,097,121,946	96.58	16	4,075,796	37,929,866	47.45	Ascomycota	*Aspergillus*	*Aspergillus* sp. Gbtc 2	–	–

*F1 and F2 belong to the Kingdom Fungi. The highly accurate long-read sequences produced on the PacBio Sequel Systems were used to assemble the draft genomes. The pair-ended data (listed in this table) were added in the polish process after the assembly. **The Completeness and Contamination was calculated in the checkM program. “–” means the non-applicable items for checkM analyses.

### Sequencing, Assembly, and Annotation

As the detailed information listed in Table 1, a total of 207,584,048 clean reads (BGISEQ-500, 2 × PE150) was obtained to include 31,137,607,200 clean bases for bacteria isolates. Their average Q20 was 94.59%, ranging from 91.20 to 97.55%, indicating the high accuracy guarantee for assembly. As to the two fungi, 848,064 and 1,791,961 sequence entries were collected with a total bases of 9,678,732,196 and 4,017,770,803 bp. As expected, the average sequence lengths were above 1K bases (11,412 and 2,242 bp, respectively).

Influenced by splicing and assembly parameters, and the species characteristics, 23 bacterial draft genomes were built with various scaffolds (from 370 to 2854). The N50 sizes varied from 58,194 to 2,312,843 bp. Meanwhile, the draft genomes sizes were from 2,604,457 to 12,471,597 bp. Comparing with the GeneBank genome database, all these draft genomes had similar genome sizes to their neighborhood species. All the sequencing depths we calculated were far more than 50×, which was enough for assembling high-quality genomes in most software (Desai et al., 2013). Undoubtedly, the overall G + C content of draft genomes varied with the phylogenetic relationship. The Phylum Actinobacteria species had higher G + C content than others, up to 75.09%, while the Firmicutes species own comparatively lower G + C content. Besides, the Proteobacteria species also possessed high G + C content. *Deinococcus* sp. Gbtc 1 (ID: B9, 68.85% G + C) seemed to verify the fact that the genus *Deinococcus* may have high G + C content (McDonald, 2001) as reported. Moreover, it is reasonable to confirm that most genes are included in the current draft genomes because the completeness estimates were all above 98.7%, while the contamination was all less than 0.16%.

Considering that fungi generally have a larger genome than bacteria, the introduction of the Single Molecule, Real-Time (SMRT) Sequencing technology is necessary to improve the assembly results, especially for eukaryotes. Here, coupled with the pair-ended PE150 reads, the Pacbio output was assembled into two high-quality draft genomes of the *Aspergillus* (16 and 18 contigs). *Aspergillus* sp. Gbtc 1 (ID: F1) possessed a 29,114,197 bp genome with a N50 of 3,752,118 bp, while *Aspergillus* sp. Gbtc 2 (ID: F2) had a genome of 37,929,866 bp with a 4,075,796 bp N50. Both had a G + C content of less than 50%, similar to these existed strains in the GeneBank database. On the other side, the BUSCO completeness was 74.9 and 74.5%, which was as large as three strains of the closest phylogeny (Supplementary Figure 2), showing the assemblies’ acceptability.

### Phylogenetic Analysis

**FIGURE 2:**
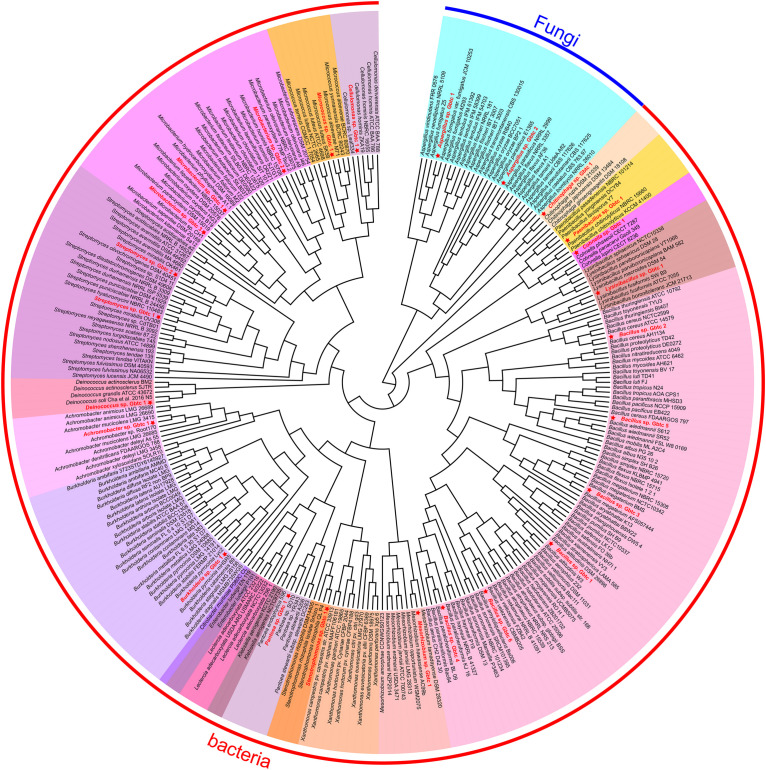
The phylogenetic tree of all isolates and other open-access strains based on the whole genomes. The red font strains with five-pointed stars ahead indicated the *Ginkgo* isolates. The same genus individuals were backgrounded with the same color. The arc-shaped line segment on the outermost circle represented the Kingdom of bacteria and fungi.

Generally, the thread of 97% 16s rRNA similarity was proposed as a golden criterion in bacterial classification for many years (Stackebrandt and Goebel, 1994). However, the dataset is becoming broader and broader, reducing the discrimination of 16s rRNA with high conservation significantly. Whereafter, a series of other genes were chosen as the variable markers in succession, such as gyrB (Yamamoto and Harayama, 1995), recA/B (Pascual et al., 2010), and *rpo*D (Parkinson et al., 2011). Nevertheless, different organisms are not consistent in the heterogeneity of these housekeeping genes (Yamamoto and Harayama, 1998; Wang et al., 2007). In this research, CVTree3 used the whole genomes of all isolates and the items from the open-access database to infer a more accurate phylogenetic tree. Unlike a single marker gene, namely 16s rRNA etc., simultaneous analyzing all bacterial and fungal genomes exhibits another advantage that all results can be presented in a phylogenetic tree (Figure 2). Due to 16s rRNA and ITS pre-classification, almost all strains were assigned into diverse clades properly, for instance, in fungi, *Aspergillus* sp. Gbtc_1 was likely to be phylogenetically affiliated to the species *Aspergillus fumigatus*, while *Aspergillus* sp. Gbtc_2 might be a member of the species *Aspergillus flavus* or *Aspergillus parasiticus* alternatively. Additionally, a similar situation was observed in the bacterial Kingdom: 13 strains were severally affiliated to multiple species seemingly, including *Cellulomonas hominis*, *Streptomyces hyaluromycini*, *Chitinophaga rupis*, *Bacillus cereus*, *Bacillus megaterium*, *Bacillus paralicheniformis*, *Bacillus velezensis*, *Lysinibacillus fusiformis*, *Paenibacillus chitinolyticus*, *Achromobacter mucicolens*, *Burkholderia pyrrocinia*, and *Stenotrophomonas rhizophila*. In fact, species identification and classification are not absolutely unique, but only reflect relative phylogenetic relationships, as we all know.

### Functional Annotation and Classification

These indicators aforementioned suggested that the 25 draft genomes we assembled here were suitable for further bioinformatics analysis. The two fungi own significantly more CDSs, rRNAs, and tRNAs than most bacteria (Supplementary Table 2). Two *Streptomyces* (**B6** and **B7**) strains expressed much larger genomes with ≥ 10,000 CDSs than other bacteria. The genus *Burkholderia* also appeared to be a large genome (∼8 Mb with 7126 CDSs) owner in the Bacteria Kingdom. Of all the databases used in this study, the Nr database had the highest percentage of similar entries. It is noteworthy that high gene annotation ratios were obtained in function prediction, which were all above 97%. However, as the host, the *Ginkgo* genome’s annotation rate is only 68.12% (Guan et al., 2016), revealing unknown species-specific genes in the *Ginkgo* genome.

**FIGURE 3:**
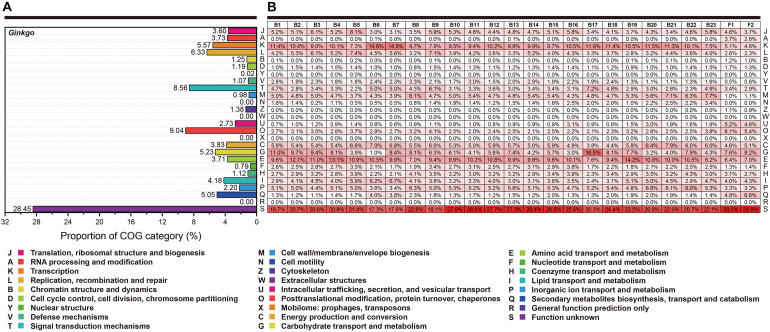
The functional categories based on the COG annotation. Each row represents the same functional classification. **(A)** The COG categories of *Ginkgo biloba* genes. **(B)** The COG categories of genes for all individual strains. The red color density indicated the percentage of genes in this functional category to the total genes annotated in each species’ COG database. At the same time, these percentage values (keep one decimal place) were marked in every square grid.

Here, we performed the COG classification to survey the functional profile. The annotation rate varied from 76.82 to 91.13%, and all annotated genes were separated into 26 groups (Figure 3). Overall, irrespective of the category “Function unknown (S),” relatively more genes belonged to “Transcription (K),” “Amino acid transport and metabolism (E)” and “Carbohydrate transport and metabolism (G)” for most bacteria. Signally, *Cohnella* sp. Gbtc 1 possessed 16.8% annotated genes belonging to the category “Carbohydrate transport and metabolism (G),” seemingly revealing its more robust carbon metabolism. However, the situation of the fungi was significantly different. The RNA process (A) and the biosynthesis of the secondary metabolites (Q) was much more prosperous. For *Ginkgo*, the categories “Signal transduction mechanisms (T)” and “Posttranslational modification, protein turnover, chaperones (O)” exhibited higher gene portion, as well as “Secondary metabolites biosynthesis, transport and catabolism (Q).” Summarily, within this regional *Ginkgo* roots (we can regard it as a host-parasite community), bacteria tended to pay more attention to primary metabolism. Simultaneously, fungi and the host *Ginkgo* have stronger secondary metabolism, which may be closely related to the host’s environmental tolerance.

### Secondary Metabolism

Generally, microorganisms may promote the secondary metabolism by participating directly or indirectly in their hosts (Hassan and Mathesius, 2012; Gluck-Thaler and Slot, 2015), even if some secondary metabolic processes only occur in the eukaryotic field. In detail, elicitors and metabolic precursors are the primary forms for plant endophytes. So, in *Ginkgo*, not only its secondary metabolites contribute to its tolerance to the abiotic and biotic stresses, mainly containing flavonoids and terpenoid lactones (Guan et al., 2016), but also there may be a series of endophytes playing critical roles in these processes through multiple synergistic effects. Therefore, a variety of secondary metabolic pathways in the *Ginkgo* root community are analyzed in this study.

#### Flavonoid Biosynthesis

**FIGURE 4:**
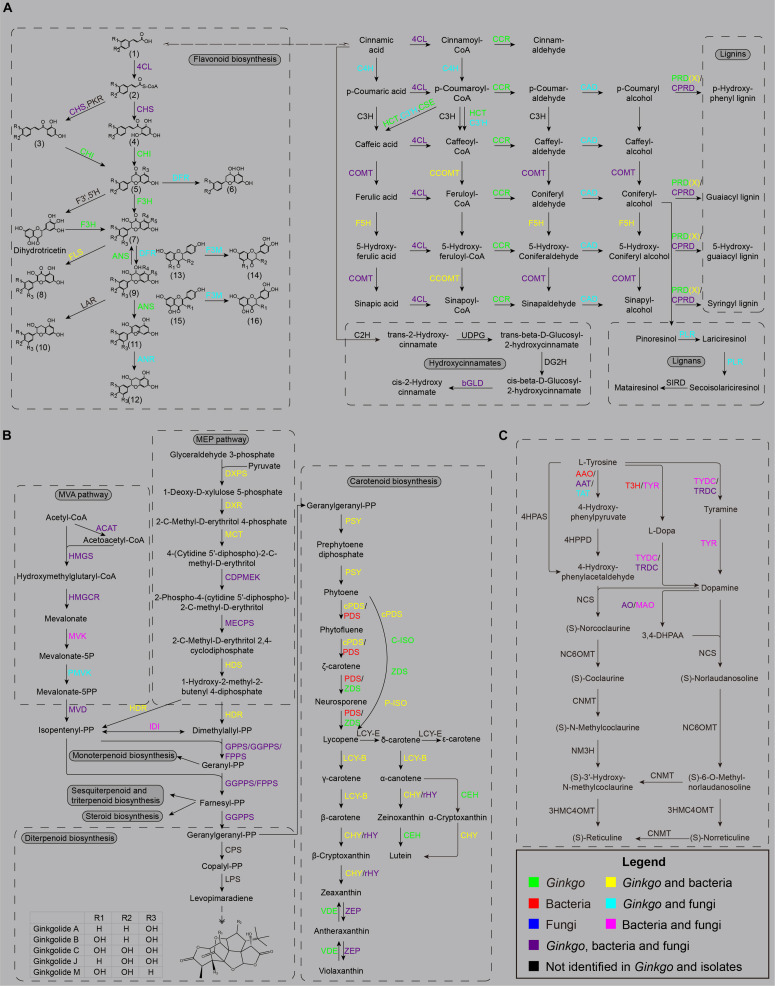
The main secondary metabolism pathways. **(A)** The biosynthesis sketch of flavonoid and non-flavonoid polyphenolics in the *Ginkgo* root community. The R labels represent a hydrogen atom, a hydroxyl, or a methoxy in all chemical structures. (1) *trans*-cinnamic acid; *p*-coumaric acid; caffeic acid; ferulic acid; (2) cinnamoyl-CoA; *p*-coumaroyl-CoA; caffeoyl-CoA; (3) isoliquiritigenin; butein; (4) pinocembrin chalcone; naringenin chalcone; eriodictyol chalcone; (5) pinocembrin; liquiritigenin; butin; naringenin; eriodictyol; (6) apiforol; luteoforol; (7) pinobanksin; garbanzol; fustin; dihydrokaempferol; dihydroquercetin; dihydromyricetin; (8) galangin; kaempferol; quercetin; myricetin; (9) 5-deoxyleucopelargonidin; 5-deoxyleucocyanidin; *cis*-3, 4-leucopelargonidin; leucocyanidin; leucodelphinidin; (10) afzelechin; (+)-catechin; (+)-gallocatechin; (11) pelargonidin; cyanidin; delphinidin; (12) (–)-epiafzelechin; (–)-epicatechin; (–)-epigallocatechin; (13) liquiritigenin; garbanzol; naringenin; dihydrokaempferol; (14) butin; fustin; eriodictyol; dihydroquercetin; (15) apigenin; kaempferol; (16) luteolin; quercetin. **(B)** The biosynthesis sketch of terpenoid backbone and their derivatives in the *Ginkgo* root community. **(C)** The biosynthesis sketch of isoquinoline alkaloid backbone in the *Ginkgo* root community.

The whole pathway of flavonoid biosynthesis is relatively complete in the *Ginkgo* genome (Figure 4A). The first regulatory step of flavonoid biosynthesis was cinnamoyl-CoA’s transformation to pinocembrin chalcone by chalcone synthase (CHS), which can also catalyze *p*-coumaroyl-CoA to naringenin chalcone, caffeoyl-CoA to eriodictyol chalcone, and feruloyl-CoA to homoeriodictyol chalcone. A total of 17 genes were putatively annotated as CHS genes, including one belonged to Sample **B6**, two belonged to Sample **F2**, and the rest belonged to the *Ginkgo* host. However, no putative genes were identified to code chalcone isomerase (CHI) and flavanone 3-hydroxylase (F3H) in all endophytes. Herein, CHI is responsible for converting various chalcones into flavonoid monomers (pinocembrin, liquiritigenin, butin, and naringenin), while F3H catalyzes these flavonoid monomers into dihydroflavonols. Notably, both *Ginkgo* and the fungi **F1** potentially possess the ability to reduce dihydroflavonols to the precursors of anthocyanin synthesis (viz. *cis*-3,4-leucopelargonidin, leucocyanidin, and leucodelphinidin), as 17 and 1 copy of dihydroflavonol 4-reductase (DFR) genes were identified in their genomes, respectively. After dehydrogenation and dehydration by anthocyanidin synthase (ANS), these precursors were catalyzed into the colorful anthocyanin class of flavonoids (cyanidin, delphinidin, pelargonidin). The functional annotation indicated that only *Ginkgo* involved 8 ANS genes in the genome. Additionally, 5 *Ginkgo* genes and 1 **F1** gene were defined as anthocyanidin reductase (ANR) encoders. More prominently, up to 54 genes (47 of *Ginkgo*, 3 of F1, and 4 of F2) encode the flavonoid 3′-monooxygenase (F3M), indicating potential frequent collaborative transformation among various flavonoids like kaempferol and quercetin. So, the host *Ginkgo* may play a major role in the synthesis of flavonoids in this root community, under the assist of several putative genes originated from bacteria (**B6** and **B8**) and these two fungi.

#### Non-flavonoid Polyphenolics Biosynthesis

More and more polyphenolic compounds are being investigated for their potential application in the pharmaceutical industry. They are always constructed from cinnamic acids via a series of condensation reactions in phenylpropanoid biosynthesis. As a major precursor, the cinnamic acid is derived from the phenylalanine and leading into flavonoids, monolignols, lignins, and lignans, while it also branch off into hydroxycinnamates (Zou et al., 2019). Therefore, these polyphenolics’ biosynthesis is based on the shared substrates, e.g., *p*-coumaroyl-CoA and feruloyl-CoA, inevitably leading to the existence of competition.

In addition to the flavonoid metabolism, other genes encoding crucial enzymes were observed in several bacterial and fungal strains, putatively participating in the biosynthesis of non-flavonoid phenolic substances in this *Ginkgo* root community. In the phenylpropanoid biosynthesis, the cinnamate 4-hydroxylase (C4H) hydroxylates the cinnamic acid or cinnamoyl-CoA into *p*-coumaric acid or *p*-coumaroyl-CoA, herein 13 and 1 copy of its encoder genes were located in *Ginkgo* and **F1** genomes. Then, the synthesis of caffeic acid and caffeoyl-CoA is accomplished through the coordination of shikimate *O*-hydroxycinnamoyltransferase (HCT), coumaroylquinate 3′-monooxygenase (C3’H) and caffeoylshikimate esterase (CSE) from their precursor *p*-coumaroyl-CoA. Three C3’H genes were observed in Sample **F2**, potentially assisting the host to complete this critical process. Two kinds of methyltransferase, namely caffeic acid 3-*O*-methyltransferase (COMT) and caffeoyl-CoA *O*-methyltransferase (CCOMT), were responsible for the transformation of caffeic acid (or caffeoyl-CoA, caffeic aldehyde, caffeyl alcohol) to ferulic acid (feruloyl-CoA, coniferyl aldehyde, coniferyl alcohol), as well as from 5-hydroxyferulic acid (or 5-hydroxyferuloyl-CoA, 5-hydroxyconiferaldehyde, 5-hydroxyconiferyl alcohol) to sinapic acid (or sinapoyl-CoA, sinapoyl aldehyde, sinapyl alcohol) in the downstream of metabolism. In this course, 19 COMT (CCOMT) isoforms were identified in the bacteria and fungi. Meanwhile, 23 genes were predicted to code ferulate-5-hydroxylase (F5H), with one belonged to bacterial strain **B6**. In the other direction, the downstream derivatization of these precursor acids is accomplished by 4-coumarate-CoA ligase (4CL), cinnamoyl-CoA reductase (CCR), and cinnamyl-alcohol dehydrogenase (CAD) in turn, to produce *p*-coumaryl alcohol, caffeyl alcohol, coniferyl alcohol, 5-hydroxyconiferyl alcohol, and sinapyl alcohol. These three enzymes were all putatively encoded in the *Ginkgo* host, while only five 4CL genes from three bacteria strains were annotated, and the two fungi strains involved both 4CL and CAD isoforms. The current favored viewpoint endorses that *p*-coumaryl alcohol, coniferyl alcohol, 5-hydroxyconiferyl alcohol, and sinapyl alcohol are the direct precursors of the lignins (*p*-hydroxyphenyl lignin, guaiacyl lignin, 5-hydroxy-guaiacyl lignin, and syringyl lignin). These monolignol oxidation occurs through peroxidase (PRD), peroxiredoxin (PRDX), or catalase-peroxidase (CPRD), thereof *Ginkgo* genome containing all annotated PRD genes and two PRDX genes while several bacterial and fungal strains affording 9 CPRD genes and one PRDX gene. As for the lignans biosynthesis, a pivotal branchpoint enzyme named pinoresinol/lariciresinol reductase (PLR) were detected in *Ginkgo* and Fungus **F1**; nevertheless, the ones involved in the downstream derivation, like secoisolariciresinol dehydrogenase (SIRD), pluviatolide synthase, pluviatolide 4-*O*-methyltransferase, bursehernin 5′-monooxygenase, 5′-demethylyatein 5′-*O*-methyltransferase, and deoxypodophyllotoxin synthase, have not been observed in all organisms.

#### Terpenoid Backbone and Their Derivatives Biosynthesis

Terpenoid is a class of the most abundant secondary metabolites in nature, wildly produced in the bacteria, fungi, and higher plants. More than 50 thousands of terpenoids have been discovered to be biologically active ingredients, comprising monoterpenes, sesquiterpene, diterpene, triterpene, and tetraterpenes (Zhang et al., 2017a). As a good model for diterpene research in higher plants, *Ginkgo* is well known for its wealthy diterpenoids of ginkgolides and bilobalide (Maclennan et al., 2002), and markedly recommended as the therapeutic usage to antagonize platelet-activating factor (PAF). Therefore, it is meaningful to thoroughly excavate the genes related to terpene synthesis to study all species’ mutual evolution and synergy in this community.

In general, all terpenoids are derived from two isomeric precursors, isopentenyl diphosphate (IPP) and dimethylallyl diphosphate (DMAPP). IPP and DMAPP are synthesized in the mevalonic acid (MEV) pathway and the 2-*C*-methyl-D-erythritol-4-phosphate (MEP) pathway (Lichtenthaler, 1999; Lange et al., 2000). In the MEV pathway (Figure 4B), three acetyl-CoA molecules are condensed and transformed into MEV and then subjected to multiple steps of phosphorylation and decarboxylation reactions to produce IPP. The MEP pathway is started with the combination of glyceraldehyde 3-phosphate and pyruvate into 1-deoxy-D-xylulose 5-phosphate, and then further catalyzed to MEP. It is then formed into 2-*C*-methyl-D-erythritol-2,4-cyclodiphosphate (MEC) through three successive reactions before MEC is further catalyzed to obtain a mixture of IPP and DMAPP of 5:1. Besides, IPP and DMAPP can be isomerized to each other with isopentenyl diphosphate isomerase (IDI). As basic C_5_ units, alternative assembly of two, three, or four these units via prenyltransferases may yield geranyl diphosphate (GPP), farnesyl diphosphate (FPP) and geranylgeranyl diphosphate (GGPP), which serve as the immediate precursors for diverse monoterpenoids (C_10_), sesquiterpenoids (C_15_), diterpenoids (C_20_), triterpenoids (C_30_), and tetraterpenoids (C_40_). In this study, a nearly integrated pathway of terpenoid backbone biosynthesis was observed in the root community. As well as the *Ginkgo* host, almost all bacterial strains were capable of producing IPP and DMAPP through the MEP pathway via 1-deoxy-D-xylulose-5-phosphate synthase (DXPS), 1-deoxy-D-xylulose-5-phosphate reductoisomerase (DXR), 2-*C*-methyl-D-erythritol 4-phosphate cytidylyltransferase (MCT), 4-diphosphocytidyl-2-*C*-methyl-D-erythritol kinase (CDPMEK), 2-*C*-methyl-D-erythritol 2,4-cyclodiphosphate synthase (ME), (*E*)-4-hydroxy-3-methylbut-2-enyl-diphosphate synthase (HDS) and 4-hydroxy-3-methylbut-2-en-1-yl diphosphate reductase (HDR). Although a widely accepted view indicates that the plant host may inherit genes in the MEP pathway from a prokaryote endosymbiont (Vranova et al., 2013), more evidence needs to be investigated in this community. The MEP pathway may probably take place in the plastids of *Ginkgo*. It is worth noting that *Aspergillus* sp. Gbtc_2 (**F2)** was discovered to hold a contiguous gene unit of CDPMEK and MECPS gene in its genome.

On the other side, the MVA pathway reconstruction indicated that strains **B1**, **F1**, **F2** and *Ginkgo* presumably facilitated the synthesis from acetyl-CoA to IPP cooperatively. Excluding errors in gene prediction and functional annotation, the *Ginkgo* host may lose the capacity to produce mevalonate-5P due to a lack of mevalonate kinase (MVK); only **B1**, **F1,** and **F2** each possessed one copy of the MVK gene. Intriguingly, **B1** (putatively affiliated to gram-positive *Cellulomonas hominis*) became the unique strain in all endophytes to retain both MEP and MVA pathways to produce IPP and DMAPP. Additionally, 99 copies of the acetyl-CoA C-acetyltransferase (ACAT) gene were observed, most belonging to bacterial strains (Supplementary File 2). The genes of hydroxymethylglutaryl-CoA synthase (HMGS), hydroxymethylglutaryl-CoA reductase (HMGCR), phosphomevalonate kinase (PMVK), and diphosphomevalonate decarboxylase (MVD) were mainly discovered in *Ginkgo* and fungal genomes.

A series of GPPS, FPPS, and GGPPS genes were identified in the downstream pathway, encoding synthases to transform IPP and DMAPP into GPP, FPP, and GGPP. Only one GPPS gene from *Ginkgo* was scanned out, while 47 and 10 CDSs were annotated to be GGPPS and FPPS genes, respectively. Remarkably, the KEGG results depicted that FPPS genes were classified into two groups (K00787 and K00795), and GGPPS genes were classified into three groups (K00804, K13787, K13789). These sequences were significantly different between eukaryotes and prokaryotes, and K13789 contained the genes from *Ginkgo* and endophytic bacteria, in which there may be close homology.

However, the pathway regeneration exhibited an incomplete biosynthesis of monoterpenoid with only three enzyme genes [K15095 (+)-neomenthol dehydrogenase, K12467 myrcene/ocimene synthase, K07385 1,8-cineole synthase]. As to the biosynthesis of sesquiterpenoid and triterpenoid, one and two CDSs were found closest to the (+)-alpha-barbatene/beta-chamigrene/thujopsene synthase and NAD+-dependent farnesol dehydrogenase genes, which are responsible for changing FPP to sesquiterpenoids, like (+)-alpha-barbatene, (+)-beta-chamigrene, and (+)-thujopsene. Moreover, farnesyl-diphosphate farnesyltransferase and squalene monooxygenase were discovered to retain genes in both *Ginkgo* and the two fungi, to transfer the farnesyl of FPP to the triterpenoid precursors, squalene and (S)-squalene-2,3-epoxide. Meanwhile, one alarm was the presence of squalene-hopene/tetraprenyl-beta-curcumene cyclase genes in bacteria and fungi rather than *Ginkgo*, which made us concerned with the derived bridge of the endophytes.

Considering the predominance of ginkgolides in *Ginkgo*, we interestedly discussed the diterpenoid biosynthesis here. Initiated by the protonation from GGPP under the copalyl diphosphate synthase (CPS), the copalyl diphosphate (CPP) was further catalyzed into levopimaradiene after allylic diphosphate ionization, cyclization, hydride shift, methyl migration, and deprotonation (Schwarz and Arigoni, 1999). Nevertheless, there were no CPS or levopimaradiene synthase (LPS) genes found in all genomes. Reciprocally, acting as a critical kind of hormones throughout the plant life-cycle, gibberellins (GA) harbored a continuing pathway in the host, which is a matter of course. Thereof, the genes of ent-copalyl diphosphate synthase and ent-kaurene oxidase occupied the largest number of isoforms in *Ginkgo*’s genome. Intriguingly, one ent-kaurenoic acid monooxygenase gene from **F1**, one gibberellin 2beta-dioxygenase gene from B6, and three gibberellin-44 dioxygenase genes from **F2** were also involved in this community, potentially facilitating and regulating the biosynthesis of GAs.

Carotenoids are 40-carbon tetraterpenoids condensed and derived from two GGPP molecules by diverse critical enzymes, including phytoene synthase (PSY), phytoene desaturase (PDS), zeta-carotene desaturase (ZDS), and others. Herein, we observed a two-approach process from phytoene to lycopene in this community: (1) cPDS/PDS - ZDS; (2) cPDS – C-ISO – ZDS – P-ISO. Subsequently, the lycopene is always converted into two branches in the downstream. One is that lycopene β-cyclase (LCY-B) transformed the parent molecule to γ-carotene and then to β-carotene in a two-step reaction, whereas the other is that lycopene ε-cyclase (LCY-E) catalyzed it into δ-carotene and ε-carotene, in which the δ-carotene is further catalyzed to α-carotene and lutein (Hirschberg, 2001). However, from an overall perspective, gene annotation depicted a carotene transformation way pointing to the xanthophyll cycle in this community. Although LCY-B, beta-carotene 3-hydroxylase (CHY), and beta-ring hydroxylase (rHY) each were capable of acting on these two branches, and one carotenoid epsilon hydroxylase (CEH) gene was harbored in the *Ginkgo* genome, the lack of LCY-E may lead to the failure in downstream biosynthesis of δ-carotene ε-cyclase, α- carotene and even lutein.

#### Isoquinoline Alkaloid Backbone Biosynthesis

As a group of nitrogen-containing natural products, alkaloids exist in approximately 20% of plant species, like Opium poppy and Madagascar periwinkle (Facchini and De Luca, 2008). In another aspect, a remarkable number of endophytes were speculated to produce distinct classes of alkaloids to exert effects on pathogenic fungi, insects, and birds (Schardl et al., 2004; Fuchs et al., 2017). Not coinciding with other secondary metabolites, various alkaloid categories are unrelated in terms of synthesis and derivation. However, common patterns have become apparent in indole alkaloid or isoquinoline alkaloid (morphine, sanguinarine, etc.). As aromatic-L-amino-acid/L-tryptophan decarboxylase (K01593) became the sole enzyme we identified in the indole alkaloid biosynthesis of this root community; detailed insight was excavated to the biosynthesis of isoquinoline alkaloid.

Notoriously, isoquinoline alkaloid biosynthesis begins with the transformation from tyrosine to dopamine and 4-hydroxyphenylacetaldehyde (4HPAA) through decarboxylation, hydroxylation, and deamination (Sato et al., 2007). The norcoclaurine synthase (NCS) condenses dopamine and 4HPAA to yield (*S*)-norcoclaurine (Facchini, 2001). Then, the norcoclaurine is converted to coclaurine by norcoclaurine 6-*O*-methyltransferase (NC6OMT), sequentially followed by a series of reactions to *N*-methylcoclaurine, 3′-hydroxy-*N*-methyl coclaurine and reticuline by coclaurine *N*-methyltransferase (CNMT), *N*-methylcoclaurine 3′-monooxygenase (NM3H), and 3′-hydroxy *N*-methylcoclaurine 4′-*O*-methyltransferase (3HMC4OMT).

Available data suggested strict reaction specificities of NC6OMT, 3HMC4OMT, and CNMT (Morishige et al., 2000), suggesting that the pathway is optional between the norcoclaurine branch and the norlaudanosoline branch before the central intermediate reticuline is ready for the next biosynthesis of isoquinoline alkaloid. In this study, we only observed the early part of the backbone biosynthesis (Figure 4C). A lack of 4-hydroxyphenylpyruvate decarboxylase and 4-hydroxyphenylacetaldehyde synthase may lead to the deficiency of 4HPAA in this root tissue, although a few aspartate aminotransferases or the monofunctional tyrosine aminotransferase (K00815) were annotated to potentially catalyze L-tyrosine to 4-hydroxyphenylpyruvate. On the other branch, the process from tyrosine to dopamine is relatively integrated in this unit. *Ginkgo* genome was constructive to harbor 6 CDSs of tryptophan decarboxylase (TRDC) and 13 CDSs of primary-amine oxidase (AO); these two proteins were also found in bacteria and fungi. The tyrosine 3-hydroxylase (T3H) in **B21** and **B23** were responsible for converting L-tyrosine into L-dopa, while the tyrosinase (TYR) in **B6**, **B7**, **B8**, **B12**, and **F2** catalyze both L-tyrosine into L-dopa and tyramine to dopamine. Additionally, as well as TRDC, the tyrosine decarboxylase (TYDC) was putatively included in the genomes of **B7**, **B8**, **F1,** and **F2**, to accomplish the transmission from L-tyrosine to tyramine and L-dopa to dopamine. Noteworthily, the manufacture of dopamine is likely dependent more on endophytes rather than the *Ginkgo* host, especially the strains **B7** (named as *Streptomyces* sp. Gbtc_2) and **F2** (named as *Aspergillus* sp. Gbtc_2). So, it can be inferred that the synthesis of dopamine is achieved through the cooperation of endophytes and the *Ginkgo* host to a certain extent, although it is impossible to put together all genomes to compose an entire metabolic pathway of isoquinoline alkaloid biosynthesis.

### Repetitive Sequences and Transposable Element

The endophytes’ genomes were estimated to comprise various repetitive sequences (Supplementary Table 3). Using the *de novo* method, from 2.15 to 17.14% of the assembled genomes were masked as transposable elements and tandem repeats. Visibly, most bacterial strains seemed to comprise a larger proportion of repetitive sequences than the fungal ones. Numerically, *Aspergillus* sp. Gbtc 2 (**F2**) covered a lower repeating sequence ratio than *Aspergillus* sp. Gbtc 1 (**F1**), although it had a much larger genome, which may be derived from its colossal gene content. Apart from the common simple repeats, the retroelements and the DNA transposons accounted for the central part of repetitive sequences. *Streptomyces* sp. Gbtc 1, Gbtc2 and *Mesorhizobium* sp. Gbtc 1 (**B6**, **B7, B19**) harbored more DNA transposons (≥450 sequences) than other bacterial endophytes. Of the retroelements, the LTR elements revealed a majority against the SINEs and LINEs. Noteworthily, *Bacillus* sp. Gbtc 2, Gbtc 3, Gbtc 5, and *Aspergillus* sp. Gbtc 2 (**B11**, **B12**, **B14**, **F2**) embraced relatively more prosperous SINEs than others. Further on, of the LTR elements, the *Ty3/Gypsy* superfamily was ubiquitous in all isolated individuals, while the *Ty1/Copia* superfamily was only detected in *Aspergillus* sp. Gbtc 1.

**FIGURE 5:**
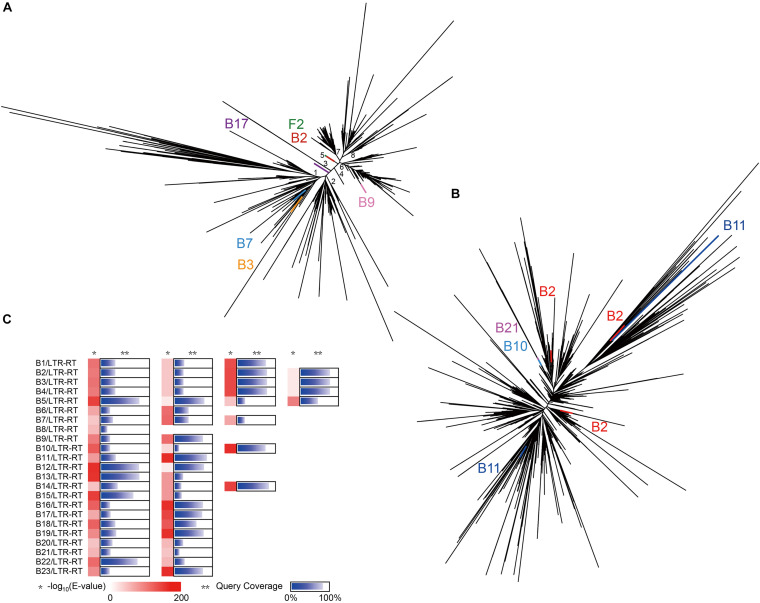
Evolution of LTR-RTs in all isolates and *Ginkgo*. **(A)** The phylogenetic tree of the *Ty3/Gypsy* superfamily predicted in all isolates and *Ginkgo.* The lines of the same color indicated the same endogenous strain, and the black lines indicated the sequences of the *Ginkgo* host. **(B)** The phylogenetic tree of the *Ty1/Copia* superfamily predicted in all isolates and *Ginkgo.* **(C)** The BLASTN output of CDSs predicted in all LTRs in isolates against the *Ginkgo* CDSs. The shade of red rectangles means the magnitude of the *E*-value, and the length of the blue bar indicates the coverage of the query sequence of CDSs predicted in all LTRs in isolates. Multiple cases in each row represent multiple LTR-RTs of the same endophyte.

To verify whether these two abundant superfamilies have undergone lateral gene transfer (LGT) between *Ginkgo* and the endophytes, the conserved transcriptase domains were carried out further multiple alignment. The phylogenetic tree of the *Ty3/Gypsy* superfamily (Figure 5A) inferred eight potential clades, four (Clade 2, 4, 7, 8) of which were notably observed as *Ginkgo*-specific clusters. Clade 1 and 2 displayed substantially higher diversity than other clades, possibly indicating the ancient origins of these subfamilies with diversification. Based on the *E*-value of 1E-5, six sequences exhibited high homology to these included in the *Ginkgo* host, though divided into different subfamilies. Two *Ty3/Gypsy*-like sequences from *Microbacterium* sp. Gbtc 2 and *Streptomyces* sp. Gbtc 2 (**B3** and **B7**) were clustered to Clade 1, during one of *Cohnella* sp. Gbtc 1 (**B17**) in Clade 3 and another of *Deinococcus* sp. Gbtc 1 (**B9**) in Clade 6. It is worth mentioning that *Microbacterium* sp. Gbtc 1 (B2), *Aspergillus* sp. Gbtc 2 (F2), and the *Ginkgo* host shared almost one remarkably similar sequence snippet to form a separate superfamily (Clade 5), indicating a potentially common ancestor. In the phylogenetic tree of the *Ty1/Copia* superfamily (Figure 5B), LTR-RTs from several endophytes and the *Ginkgo* host depicted a slightly different pattern that no clades were clustered to be easily distinguished. Three homologies of *Ty1/Copia* were observed in *Microbacterium* sp. Gbtc 1 (B2) within different branches, while two excerpts involved in *Bacillus* sp. Gbtc 2 (B11) emanated relatively longer evolutionary distance. Additionally, two sequences from *Bacillus* sp. Gbtc 1 (B10) and *Burkholderia* sp. Gbtc 1 (B21) overlapped well to reveal their conservatism.

Furthermore, gene exchange potential between endophytes and the *Ginkgo* host through LTR-RTs was estimated in Figure 5C. A total of 57 CDS (detail listed in Supplementary File 3) predicted from endophytes’ LTR-RTs were homologous to *Ginkgo* sequences. Generally speaking, the lower *E*-value and the higher query coverage indicate a higher potential for gene cross-species communication. Here, 16 pieces with the desired *E*-value (<1E-100) and >75% query coverage in the comparison coincided with the unique *Ginkgo* CDS (Gb_09036); nevertheless, it was annotated as a hypothetical protein. So, in terms of these manifestations, the genetic communication was probably widespread between endophytes and the host *Ginkgo*, and retrotransposons may be one form of the cross-species gene exchange.

## Discussion

### General Comparison of *Ginkgo* Endophytic Genomes

With the draft genome sequencing and analysis, we have performed the first comparative genomic study between the published *Ginkgo* genome (Guan et al., 2016) and its 25 endophytes of significant phenotypic differences. Articles reported that some endogenetic strains belonging to the genera *Streptomyces*, *Bacillus*, *Burkholderia*, and *Aspergillus* were already discussed on their biosynthesis of phenolic and flavonoid (Qiu et al., 2010), enzymes (Yuan et al., 2010), fungal inhibition (Yuan et al., 2012), and the taxonomy classification (Yan et al., 2018) in *Ginkgo*. Herein, 12 new genera strains isolated from *Ginkgo* root were first described as *Ginkgo* endophytes. Compared to the others, the GC contents of the Phylum Actinobacteria were much higher, in accordance with the paradigm that Actinobacteria are universally high-GC organisms in free-living environments (Ghai et al., 2012). Generally, a higher GC content means a higher optimal growth temperature for prokaryotes (Musto et al., 2006). Firmicute strains harbored significantly lower GC content than other Phyla, potentially representing special lineages in the endogenous environment.

The COG annotation showed a differentiated function map. The functional genes of most bacteria are mainly concentrated in the COG categories [K], [C], [G], and [E], indicating a potential metabolic dominance in transcription, energy production and conversion, carbohydrate transport and metabolism, amino acid transport and metabolism. Besides, the COG category [M] was also of higher occupancy in bacteria than the fungi; a plausible explanation is that bacteria is always smaller than fungi to require more efficient utilization of nutrients from the external environments (Zhang et al., 2017b). Notably, two Streptomyces strains (**B6** and **B7**), with a large genome (∼12 Mb, ∼11,000 CDSs), harbored more genes involved in transcription (Category [K]) than other bacterial isolates, which may be related to their higher proportion of secondary metabolic genes in Category [Q]. The proof that a large set (around 12% of the total chromosome) of regulatory genes was employed for metabolic regulation may support this inference (Romero-Rodriguez et al., 2015). However, the proportion of **B6** genes classified into Category [G] is significantly lower than the reported one (Harunari et al., 2018), although both were assumed to be *Streptomyces hyaluromycini*, which may suggest the potential of gene loss in carbohydrate transport and metabolism. As a comparison, the strain **B17**, high similar to *Cohnella phaseoli* in phylogeny, contained a prominent proportion of genes annotated in this category, although there is currently no relevant report explaining the specific reasons. These results provided a tip of the iceberg of the metagenome in *Ginkgo* roots.

### Endophytes Potentially Compensate or Share Part of Secondary Metabolism Pathways in *Ginkgo* Root

For a long time, the coexistence and evolution of endophytes and their plant host have established a special relationship, which may influence the production of active metabolites in plants (Jia et al., 2016). The past few years have witnessed the significant contribution of metabolite detection as one of the most intuitive methods in life sciences research. Nevertheless, the diversification and the complexity of the components always lead to analytical variability, ion suppression and metabolite identification, which has become the bottleneck for the metabolome study (de Jong and Beecher, 2012). Preliminary analysis based on comparative genomics may be a powerful means to simplify metabolite research, especially in the unknown field of endophyte-host study.

The biosynthesis of polyphenols is an essential branch of phenylpropanoid metabolism, usually originating from the phenylalanine conversion. Up to now, few bacterial counterparts were observed to simultaneously harbor the phenylalanine ammonia-lyase (PAL), 4CL and CHS genes, reflecting the conceivable inexistence of directly producing chalcones in bacteria (Moore et al., 2002). Here we propose a new conjecture based on our analysis results: a variety of endogenous bacteria can work together to convert phenylalanine to chalcones in a microenvironment in a complementary manner. The isolates **B21**, **B6**, and **B3,** seemingly supported our conjecture due to their inclusion of the PAL, 4CL and CHS genes in their genomes, respectively. Besides, the endogenous fungus **F2** (high homologous to *Aspergillus flavus*) harbored these three genes to expand our insights. The homologous genes of CHI was reported to exist in some fungi, slime molds and γ-proteobacteria, while these species with CHI-like genes often lack orthologous genes for the upstream enzyme CHS (Gensheimer and Mushegian, 2004), which seems to run counter to our results here. Evidences suggested that endophytic fungi can participate in their plant hosts’ flavonoid metabolism by producing new flavonol (Yan et al., 2013) or metabolizing glycosylated flavonoids into aglycone (Tian et al., 2014). In this study, endogenous fungi potentially have sufficient ability to derivatize downstream flavonoids by adding hydroxyl groups. Given the competition, most polyphenolics (flavonoids, lignins, lignans, and hydroxycinnamates) are synthesized from the same substrates (Zou et al., 2019), endophytes and the *Ginkgo* host may be involved in the regulation of material flow at the same time. As for the synthesis of lignins, there is no relevant evidence supporting the promoting effect of synthesis, yet most reports have pointed out that endophytic bacteria and fungi can accelerate lignins’ decomposition (Koide et al., 2005; Shi et al., 2015). It seems to be inconsistent with the appearance that two lignin synthetase (CPRD, PRDX) genes were found in multiple isolates, which may be the first time to uncover the possibility of promoting lignin synthesis for the endophytes parasitized in the plant. On the other side, some endophytes may directly or indirectly synthesize lignans, yet the material conversion mechanism is not precise. For instance, *Trametes hirsuta* became a novel alternative source of aryl tetralin lignans as an endophyte of *Podophyllum hexandrum* (Puri et al., 2006). The metabolites of endophytes were tended to promote the lignan synthesis in the host (Kumar et al., 2012). Herein, the sole PLR gene belonging to *Aspergillus* sp. Gbtc_1 indicated the feasibility of regulating the transformation from alcohols to lignans for *Ginkgo*, which may expand the view of the lignan synthesis field by endophytic microorganisms. Unfortunately, including the *Ginkgo* host, the synthesis pathway of hydroxycinnamates is not fully presented in this study.

Ginkgolides are an important class of active terpenoids in *Ginkgo*, which appeared to be independently biosynthesized in leaves and roots and stored in root bark and stem as hydroxylated forms (Huh and Staba, 1993). Since the LPS cDNA was first cloned and functionally characterized from *Ginkgo* (Schepmann et al., 2001), ginkgolide biosynthesis has gradually entered the public eyes. However, the authors exerted the inadequacy of LPS genes for homology comparison and analysis due to their insufficient sequence conservation (Schepmann et al., 2001; Ro and Bohlmann, 2006). This is the possible reason why this critical enzyme cannot be predicted here in the *Ginkgo* host, because its existence is indisputably actual. Like *Pestalotiopsis uvicola* GZUYX13 (Qian et al., 2016) and *Fusarium oxysporum* SYP0056 (Cui et al., 2012), multiple endophytes were isolated from *Ginkgo* trees and proved to produce ginkgolides. Although there is no reported evidence to uncover the detail of the synthesis process in these endophytes, an existing case may be able to give researchers great inspiration. The endophytes did not share the significant sequence homology of taxol biosynthetic genes with the *Taxus brevifolia*, but indicating a novel taxol biosynthesis pathway potentially independently developed in themselves (Heinig et al., 2013). Therefore, it needs further confirmation that whether the ginkgolide-producing endophytes in *Ginkgo* advocate a similar rule as the taxol-producing endophytes in *Taxus*. Nonetheless, when the mutant strain, *Aspergillus aculeatinus* BT-2, increased taxol production, the high expression of MVA pathway genes and GGPPS genes gave us favorable thinking (Qiao et al., 2020), because ginkgolides and taxol are both diterpenoid natural derivates. Also, exogenous and endogenous increase of GGPPS can increase taxol’s fungal production (Soliman et al., 2017); this evidence pushed us to think that the enhancement of MVA and MEP pathways is also very likely to promote the synthesis of active ingredients in host plants. Fortunately, the endophytic fungus *Alternaria alternata* TPF6 has been proven to increase paclitaxel synthesis by enhancing the MVA pathway and co-overexpressing heterologous IDI genes (Bian et al., 2017). In this study, the *Ginkgo* host has a complete MEP pathway to synthesize IPP and DMAPP, while IDI genes were only identified in the multiple isolates, indicating the probably key role of these endophytes in regulating the mutual transformation of these two components. If the MEP pathway of *Ginkgo* came from an endosymbiotic prokaryote as widely accepted (Vranova et al., 2013), the MVK gene involved in the original MVA pathway of *Ginkgo* might have a non-high-homology replaceable copy, or it may be compensated by endophytes due to a long-term symbiotic relationship, like *Cellulomonas* sp. Gbtc_1 (**B1**) here. Although most bacteria and the *Ginkgo* host contain a complete MEP pathway, **B1** may be the only endogenous bacteria with almost complete MEP and MVA pathways in our isolates. On the other hand, in the root environment herein, the *Ginkgo* host and endophytic bacteria may play a leading role in carotenoid synthesis. Previously, quite a few reports have confirmed that bacteria are a large natural source of carotenoids, including *Flavobacterium* spp., *Agrobacterium* spp., *Micrococcus* spp., *Chromobacterium* spp., *Rheinheimera* spp. and so on (Ram et al., 2020). Novel carotenoids have also been discovered in *Deinococcus radiodurans* and *Thermus thermophiles* to withstand extreme environmental stresses such as radiation, oxidation, and desiccation (Tian and Hua, 2010). So, given that stressful environments usually lead to the accumulation of carotenoids (Paliwal et al., 2017), we speculate that no matter whether there is material communication and regulatory interaction between endophytic bacteria and *Ginkgo* cells or not, jointly resisting environmental pressure may be their ultimate goal.

Isoquinoline alkaloids are one of the most extensive natural products in the plant kingdom, e.g., in the families of Annonaceae, Fumariaceae, Papaveraceae, and Rutaceae (Gao et al., 2008). Most of them are biosynthesized from tyrosine and show inhibitory properties against universally pathogenic fungi (Zhao et al., 2019). However, mycobionts of lichens, Streptomycetes and several fungi like *Penicillium* (Trisuwan et al., 2010) and *Aspergillus* (Kohno et al., 1999) were able to produce a few isoquinolines. They are all produced biogenetically by amination in the polyketide pathway (Yang et al., 2012). So far, there is no relevant research discussing the metabolic characteristics of *Ginkgo* endophytes in terms of isoquinoline alkaloids. Herein, no superior numbers of alkaloid-related genes were discovered, although *Ginkgo* has a solid adaptability to environmental pressure; the possible explanation is that ginkgolides and flavonoids are its main environmental tolerance guarantees, or that *Ginkgo*-specific genes constitute the unknown alkaloid synthesis pathways. Gene function mapping shows that most CDSs annotated to the alkaloid-related pathways were gathered in the isoquinoline alkaloid biosynthesis. Moreover, the large number of these copies are focused on the dopamine synthesis and the L-dopa degradation. As for as our knowledge goes, dopamine plays an important role in signal transduction in plants, and is a precursor of essential alkaloids (Lundström and Agurell, 1971). However, L-dopa has a significant two-sided effect that its considerable accumulation can increase toxicity and act as a feeding repellent, and the ROS damage generated after autooxidation cannot be ignored (Soares et al., 2014). This study may indicate that *Ginkgo* can convert L-dopa into dopamine, but its bacterial and fungal endophytes may play a potential regulatory role between these two components.

### Cross-Species Transfer of Repetitive Sequences and Genes May Occur Between the Endophytes and the *Ginkgo* Host

The cross-species exchange of genetic information has been a research hotspot for many years. The LGT, also known as horizontal gene transfer (HGT), was the most well-known form of the cross-species genetic exchange. Originated from bacteria and archaea, LGT often occurs in the phagocytic-lifestyle prokaryotes with a relatively high incidence (Ochman et al., 2000), while its situation in the eukaryotic field is more complicated. Gene transfer usually promotes eukaryotes acquiring functions from the prokaryotic genome and enables eukaryotes to settle in a new environment. The most typical example is the endosymbiotic theory (Zimorski et al., 2014). Higher-level genetic information exchange is gradually uncovered between multiple species areas, such as bacteria, fungi, animals, and plants. In general, the greater the period of the symbiotic relationship between species, the more likely LGT will occur. Therefore, we approve that the root of the longevous wild *Ginkgo* is a suitable choice for studying the genetic information exchange between higher plant hosts and endophytes. There are many ways to evaluate gene lateral transfer, but the generally accepted method is the phylogenetic tree analysis based on sequence similarity, although sometimes accuracy and universality not very appropriate (Bapteste et al., 2004).

Moran and Jarvik firstly reported that the genes responsible for the various colors of aphids were derived from the fungi (Moran and Jarvik, 2010). These genes involved in carotenoid synthesis have been integrated into the host genome, but the larger introns and larger intergenic regions were obtained in the aphid copy, obviously indicating the probable self-modification by the host. Another example, the insect host delineated the pick-up of nearly the entire genome from the endophytes *Wolbachia* that most of these sequences were non-functional fragments (Dunning Hotopp et al., 2007; Zhaxybayeva and Doolittle, 2011). Here, insertion and integration into the host genome is an important step for this process. However, it is still ambiguous that the numerous and widely distributed LTR-RTs may also be lateral gene transferred between species. Guan et al. (2016) presented phylogenetic trees of the LTR-RTs superfamilies, *Ty3/Gypsy* and *Ty1/Copia*, to show the evolutionary but not the gene transfer relationship between *Ginkgo biloba*, *Physcomitrella patens*, *Picea abies*, *Populus trichocarpa*, and *Zea may*. Therefore, we believe that gene transfer can be defined as the exchange of genetic information across very distant species, rather than the differential evolutionary succession of closely related species based on a common ancestor. As earlier reports, all LTR-RTs have surprising sequence similarities with vertebrate retroviruses (Flavell, 1999), such as the decisive fact that at least one LTR retroposon in *Drosophila* was confirmed to be an infectious retrovirus (Kim et al., 1994). This lays the foundation for our speculation that cross-species genetic exchange may also occur in the internal environment of symbiotic plants, even if the probability may be low. Our results seem to be following our conjecture that the two LTR-RT superfamily have a small number of highly similar sequences. The high sequence similarity across Kingdoms can be undoubtedly regarded as a typical LGT behavior. Moreover, dramatically, almost all isolated CDSs involved in endophytic bacteria LTR-RTs shared striking sequence similarities with the same CDS from *Ginkgo*; seemingly, LTR-RT became a rare unit flowing between the *Ginkgo* host and the endophytes to exchange genetic information. For this process, unfortunately, this CDS function cannot be estimated, and further experimental exploration is needed.

## Conclusion

This research is the first insight into the potential symbiosis-evolution relationship between the *Ginkgo biloba* and the endophytes in root. A total of 25 endophytic strains were screened and distributed in 16 genera of 6 phyla, including *Bacillus*, *Microbacterium*, *Streptomyces*, *Aspergillus*, etc. Due to the significant morphological diversities of these isolates, the functional classification profile of COG annotation shows diversified characteristics. The KEGG pathway comparison indicated that endophytes might participate in the secondary metabolism of the *Ginkgo* host in a shared or complementary manner, including the synthesis and derivation of flavonoids and terpenoids. Besides, the repetitive sequence analysis delineated a few endophytic sequences belonging to the two LTR-RT superfamilies, *Ty3/Gypsy* and *Ty1/Copia*, exhibiting extremely high similarity to that of the *Ginkgo* host. Moreover, a series of CDSs involved in such LTR-RT sequences were found highly homologous to one CDS of the *Ginkgo* host, potentially implying that LTR-RT became a rare unit flowing between the *Ginkgo* host and the endophytes to exchange genetic information. Comparative genomics is the primary analytical method, but many complex experiments are needed to verify our conjecture in this research. In short, these findings have promoted a deeper understanding of the relationship between *Ginkgo* and its endophytes, and provided a favorable reference for the subsequent development of *Ginkgo* resources and the utilization of endophytic metabolism engineering.

## Data Availability Statement

The datasets presented in this study can be found in online repositories. The names of the repository/repositories and accession number(s) can be found below: https://www.ncbi.nlm.nih.gov/bioproject/, PRJNA720764. In BioProject PRJNA720764, all assembled genomes of endophytes are available with the accession numbers SAMN18679183, SAMN18679182, SAMN18679181, SAMN18679180, SAMN18679179, SAMN18679178, SAMN18679177, SAMN18679176, SAMN18679175, SAMN18679174, SAMN18679173, SAMN18679172, SAMN18679171, SAMN18679170, SAMN18679169, SAMN18679168, SAMN18679167, SAMN18679166, SAMN18679165, SAMN18679164, SAMN18679163, SAMN18679162, SAMN18679161, SAMN18679160, and SAMN18679159. All filtered strains have been submitted to the Culture Collection Center of Key Laboratory of Biometallurgy of Ministry of Education, Central South University, China, with the accession IDs in the range CBCBSUCSU20210001-CBCBSUCSU20210025.

## Author Contributions

KZ, XL, YL, QH, and DZ conceived and designed the work. KZ, SF, and SZ performed the experiments. KZ, DZ, and QH analyzed the data. KZ wrote the manuscript. HH, FL, GZ, BM, DM, LJ, HL, and HY revised the manuscript. All the authors read, revised, and approved the final manuscript.

## Conflict of Interest

The authors declare that the research was conducted in the absence of any commercial or financial relationships that could be construed as a potential conflict of interest.

## Footnotes

1 https://www.isa-arbor.com/

2 http://www.genome.jp/kegg/kaas/
