# Supplementary material for: Root Endophytes and Ginkgo biloba Are Likely to Share and Compensate Secondary Metabolic Processes, and Potentially Exchange Genetic Information by LTR-RTs
Source: Front Plant Sci. 2021 Jul 9;12:704985. doi: 10.3389/fpls.2021.704985 (PMC8301071; doi:10.3389/fpls.2021.704985)
Supplement: Supplementary file 4 [file Data_Sheet_4.docx]

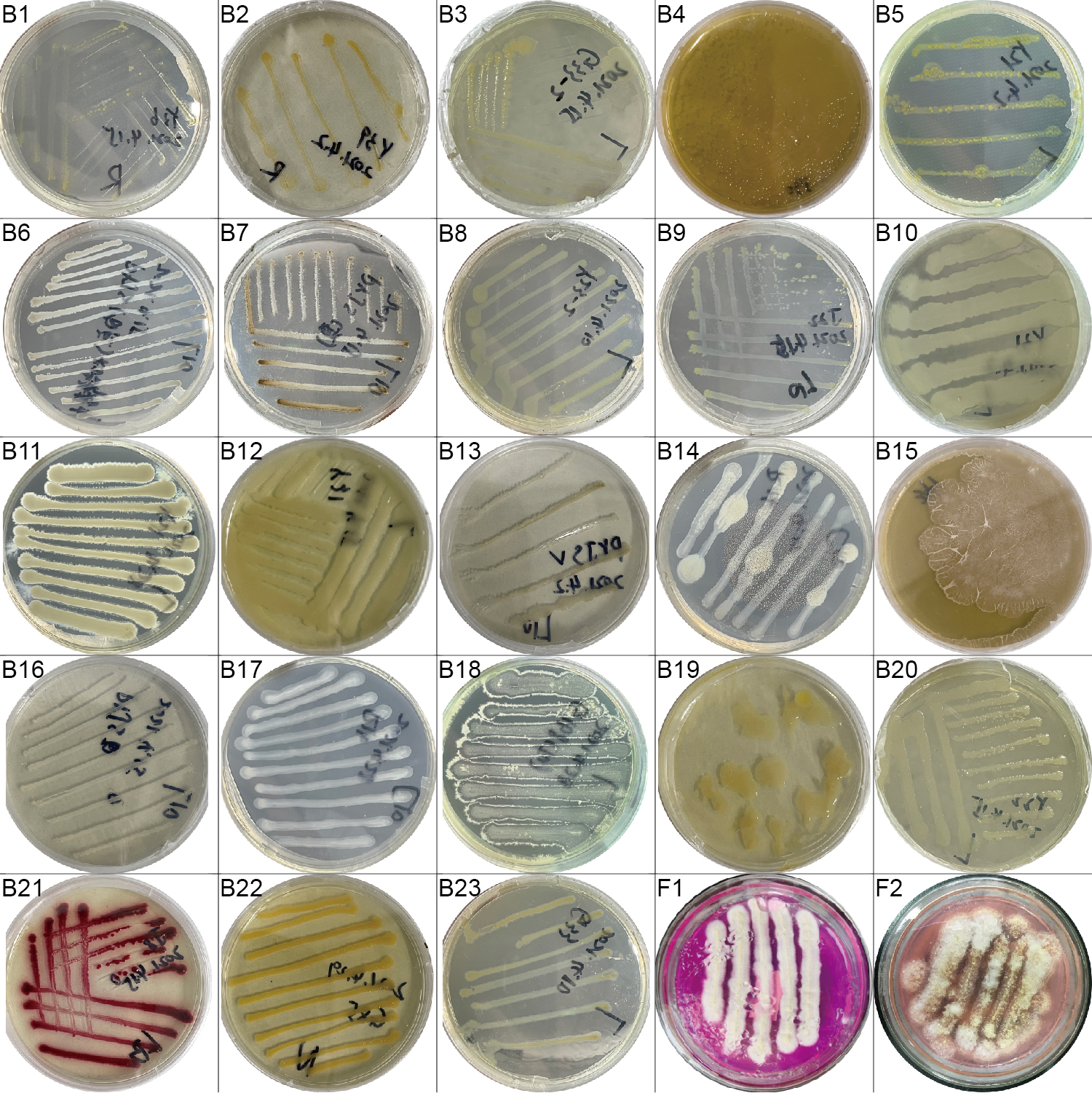


**Figure S1. The colony morphology photos of all reserved strains.**


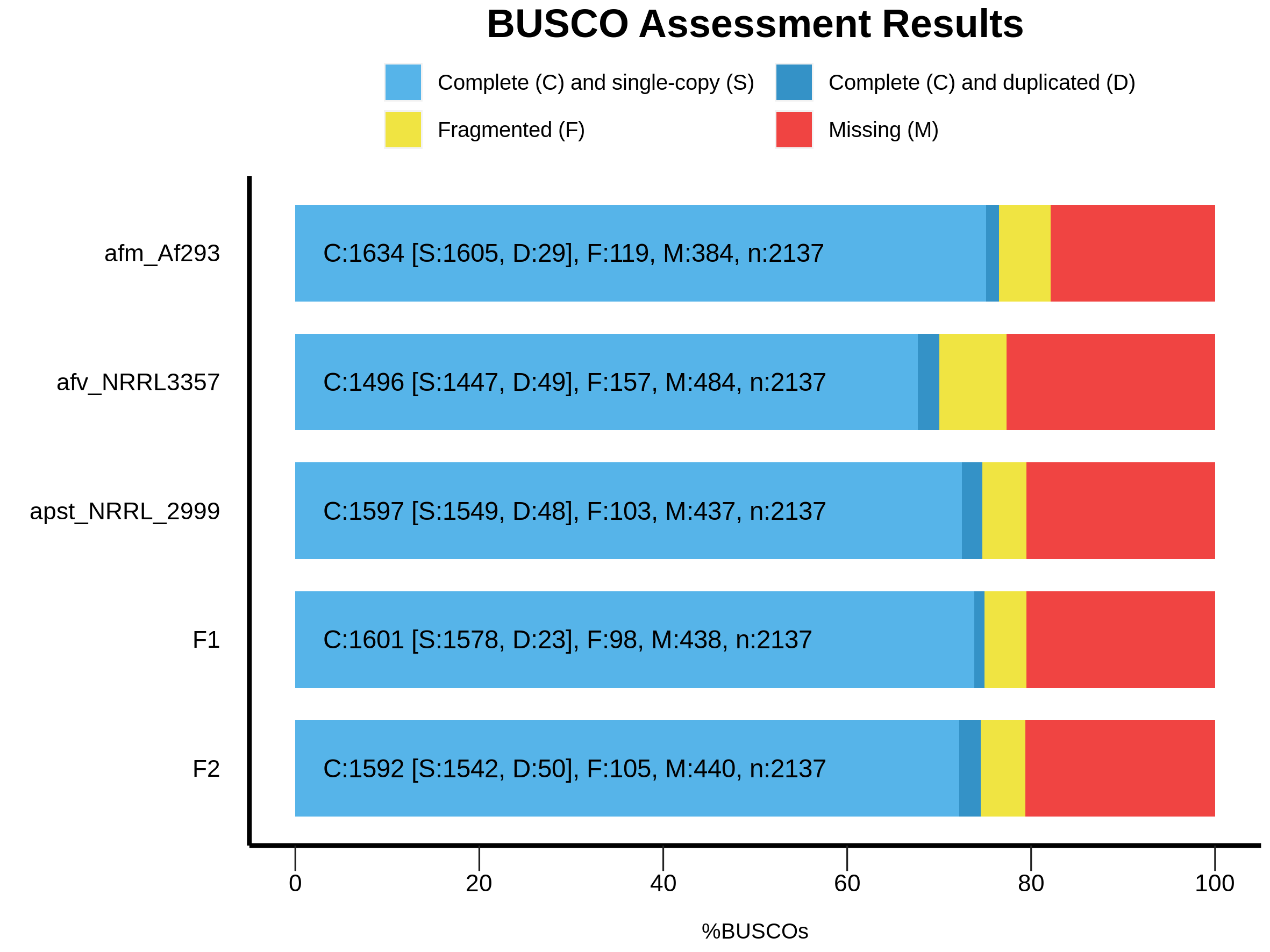


**Figure S2. BUSCO assessment of *Aspergillus* sp. Gbtc_1 (F1), *Aspergillus* sp. Gbtc_2 (F2) and other three homologous species.** “afm” represents *Aspergillus fumigatus*. “afv” represents *Aspergillus flavus*. “apst” represents *Aspergillus parasiticus*.
