# Supplementary material for: Root Endophytes and Ginkgo biloba Are Likely to Share and Compensate Secondary Metabolic Processes, and Potentially Exchange Genetic Information by LTR-RTs
Source: Front Plant Sci. 2021 Jul 9;12:704985. doi: 10.3389/fpls.2021.704985 (PMC8301071; doi:10.3389/fpls.2021.704985)
Supplement: Supplementary file 5 [file Data_Sheet_5.docx]

**Table S1. The statistics of the suspected same species.**

| ID | Phylum | Genus | Strain Name | Putative species | The No. of the suspected same species |
| --- | --- | --- | --- | --- | --- |
| B1 | Actinobacteria | *Cellulomonas* | *Cellulomonas* sp. Gbtc 1 | *Cellulomonas hominis* | 1 |
| B2 | Actinobacteria | *Microbacterium* | *Microbacterium* sp. Gbtc 1 | *Microbacterium foliorum* | 3 |
| B3 | Actinobacteria | *Microbacterium* | *Microbacterium* sp. Gbtc 2 | *Microbacterium oleivorans* | 1 |
| B4 | Actinobacteria | *Microbacterium* | *Microbacterium* sp. Gbtc 3 | *Microbacterium paraoxydans* | 3 |
| B5 | Actinobacteria | *Micrococcus* | *Micrococcus* sp. Gbtc 1 | *Micrococcus aloeverae* | 1 |
| B6 | Actinobacteria | *Streptomyces* | *Streptomyces* sp. Gbtc 1 | *Streptomyces hyaluromycini* | 2 |
| B7 | Actinobacteria | *Streptomyces* | *Streptomyces* sp. Gbtc 2 | *Streptomyces mirabilis* | 5 |
| B8 | Bacteroidetes | *Chitinophaga* | *Chitinophaga* sp. Gbtc 1 | *Chitinophaga rupis* | 3 |
| B9 | Deinococcus-Thermus | *Deinococcus* | *Deinococcus* sp. Gbtc 1 | *Deinococcus grandis* | 2 |
| B10 | Firmicutes | *Bacillus* | *Bacillus* sp. Gbtc 1 | *Bacillus altitudinis* | 1 |
| B11 | Firmicutes | *Bacillus* | *Bacillus* sp. Gbtc 2 | *Bacillus cereus* | 3 |
| B12 | Firmicutes | *Bacillus* | *Bacillus* sp. Gbtc 3 | *Bacillus megaterium* | 2 |
| B13 | Firmicutes | *Bacillus* | *Bacillus* sp. Gbtc 4 | *Bacillus paralicheniformis* | 2 |
| B14 | Firmicutes | *Bacillus* | *Bacillus* sp. Gbtc 5 | *Bacillus wiedmannii* | 3 |
| B15 | Firmicutes | *Bacillus* | *Bacillus* sp. Gbtc 6 | *Bacillus velezensis* | 3 |
| B16 | Firmicutes | *Lysinibacillus* | *Lysinibacillus* sp. Gbtc 1 | *Lysinibacillus fusiformis* | 3 |
| B17 | Firmicutes | *Cohnella* | *Cohnella* sp. Gbtc 1 | *Cohnella* sp. | 1 |
| B18 | Firmicutes | *Paenibacillus* | *Paenibacillus* sp. Gbtc 1 | *Paenibacillus chitinolyticus* | 3 |
| B19 | Proteobacteria | *Mesorhizobium* | *Mesorhizobium* sp. Gbtc 1 | *Mesorhizobium hawassense* | 2 |
| B20 | Proteobacteria | *Achromobacter* | *Achromobacter* sp. Gbtc 1 | *Achromobacter mucicolens* | 2 |
| B21 | Proteobacteria | *Burkholderia* | *Burkholderia* sp. Gbtc 1 | *Burkholderia pyrrocinia* | 2 |
| B22 | Proteobacteria | *Pantoea* | *Pantoea* sp. Gbtc 1 | *Pantoea* sp. | 1 |
| B23 | Proteobacteria | *Stenotrophomonas* | *Stenotrophomonas* sp. Gbtc 1 | *Stenotrophomonas rhizophila* | 2 |
| F1* | Ascomycota | *Aspergillus* | *Aspergillus* sp. Gbtc 1 | *Aspergillus fumigatus* | 5 |
| F2* | Ascomycota | *Aspergillus* | *Aspergillus* sp. Gbtc 2 | *Aspergillus flavus* | 7 |

The suspected same species were selectively abandoned according to the colonies' morphology and the blast results of the 16s rDNA and ITS sequences.

**Table S2. The detailed summary of gene prediction and annotation.**

| ID | B1 | B2 | B3 | B4 | B5 | B6 | B7 | B8 | B9 | B10 | B11 | B12 | B13 | B14 | B15 | B16 | B17 | B18 | B19 | B20 | B21 | B22 | B23 | F1 | F2 |
| --- | --- | --- | --- | --- | --- | --- | --- | --- | --- | --- | --- | --- | --- | --- | --- | --- | --- | --- | --- | --- | --- | --- | --- | --- | --- |
| CDS | 3922 | 3635 | 2821 | 3377 | 2258 | 10927 | 11112 | 6319 | 4103 | 3862 | 5603 | 5466 | 4276 | 5799 | 3762 | 4656 | 6559 | 5899 | 6262 | 5661 | 7126 | 4938 | 3565 | 9670 | 13307 |
| rRNA | 4 | 3 | 3 | 3 | 5 | 5 | 4 | 3 | 2 | 5 | 9 | 6 | 4 | 7 | 5 | 9 | 6 | 4 | 3 | 4 | 3 | 5 | 4 | 49 | 68 |
| tRNA | 69 | 51 | 52 | 57 | 52 | 93 | 99 | 56 | 51 | 64 | 79 | 97 | 66 | 74 | 76 | 61 | 59 | 83 | 52 | 58 | 71 | 74 | 73 | 345 | 290 |
| tmRNA | 1 | 1 | 1 | 1 | 1 | 1 | 1 | 1 | 1 | 1 | 1 | 1 | 1 | 1 | 1 | 1 | 2 | 1 | 1 | 1 | 1 | 1 | 2 | - | - |
| nr | 3847 | 3542 | 2779 | 3363 | 2239 | 10714 | 10928 | 6172 | 4021 | 3850 | 5586 | 5435 | 4260 | 5771 | 3752 | 4603 | 6415 | 5733 | 6160 | 5614 | 7065 | 4858 | 3525 | 9625 | 13216 |
| KEGG | 565 | 388 | 352 | 394 | 343 | 1308 | 1360 | 1186 | 1325 | 1107 | 1211 | 1338 | 1448 | 1230 | 2057 | 1010 | 957 | 1193 | 968 | 1084 | 1383 | 2290 | 698 | 3455 | 3902 |
| COG/KOG | 3359 | 2936 | 2427 | 2896 | 1996 | 9092 | 9149 | 5351 | 3152 | 3425 | 4685 | 4531 | 3823 | 4775 | 3399 | 3946 | 5744 | 4915 | 5422 | 5039 | 6326 | 4500 | 3112 | 8052 | 10739 |
| All^1^ | 565 | 388 | 352 | 394 | 343 | 1305 | 1358 | 1185 | 1323 | 1107 | 1211 | 1338 | 1448 | 1230 | 2042 | 1009 | 956 | 1192 | 967 | 1084 | 1383 | 2290 | 698 | 3450 | 3899 |
| at least one^2^ | 3847 | 3542 | 2779 | 3363 | 2239 | 10715 | 10928 | 6172 | 4021 | 3850 | 5586 | 5435 | 4260 | 5771 | 3752 | 4603 | 6415 | 5733 | 6160 | 5614 | 7065 | 4858 | 3525 | 9625 | 13216 |
| percentage^3^ (%) | 98.09 | 97.44 | 98.51 | 99.59 | 99.16 | 98.06 | 98.34 | 97.67 | 98.00 | 99.69 | 99.70 | 99.43 | 99.63 | 99.52 | 99.73 | 98.86 | 97.80 | 97.19 | 98.37 | 99.17 | 99.14 | 98.38 | 98.88 | 99.53 | 99.32 |

^1^ Annotated in all databases

^2^ Annotated in at least one database

^3^ Calculated on the genes annotated in all databases

**Table S3. The detailed summary of repeated sequence prediction and annotation.**

| **Sample** | **B1** | **B2** | **B3** | **B4** | **B5** | **B6** | **B7** | **B8** | **B9** | **B10** | **B11** | **B12** | **B13** | **B14** | **B15** | **B16** | **B17** | **B18** | **B19** | **B20** | **B21** | **B22** | **B23** | **F1** | **F2** |
| --- | --- | --- | --- | --- | --- | --- | --- | --- | --- | --- | --- | --- | --- | --- | --- | --- | --- | --- | --- | --- | --- | --- | --- | --- | --- |
| Bases masked (%) | 12.20 | 14.25 | 17.14 | 15.80 | 14.51 | 6.75 | 6.06 | 2.85 | 5.98 | 10.07 | 8.38 | 7.65 | 10.29 | 8.44 | 12.60 | 5.80 | 5.49 | 6.92 | 7.86 | 6.72 | 7.94 | 6.08 | 6.73 | 5.28 | 2.15 |
| Retroelements | 322 | 385 | 289 | 347 | 219 | 711 | 518 | 250 | 221 | 227 | 381 | 683 | 352 | 341 | 407 | 261 | 415 | 494 | 690 | 405 | 575 | 399 | 273 | 797 | 719 |
| SINEs | 6 | 6 | 4 | 5 | 7 | 11 | 11 | 6 | 10 | 11 | 102 | 270 | 8 | 103 | 15 | 12 | 3 | 13 | 78 | 12 | 14 | 13 | 6 | 55 | 156 |
| Penelope | 0 | 0 | 0 | 0 | 0 | 0 | 0 | 0 | 0 | 0 | 0 | 0 | 0 | 0 | 0 | 0 | 0 | 0 | 0 | 0 | 0 | 0 | 0 | 0 | 0 |
| LINEs | 1 | 16 | 0 | 1 | 0 | 11 | 2 | 70 | 0 | 8 | 19 | 0 | 23 | 6 | 23 | 14 | 0 | 31 | 6 | 0 | 3 | 4 | 1 | 50 | 15 |
| CRE/SLACS | 0 | 0 | 0 | 0 | 0 | 0 | 0 | 0 | 0 | 0 | 0 | 0 | 0 | 0 | 0 | 0 | 0 | 0 | 0 | 0 | 0 | 0 | 0 | 0 | 0 |
| L2/CR1/Rex | 0 | 0 | 0 | 0 | 0 | 0 | 0 | 0 | 0 | 0 | 0 | 0 | 0 | 0 | 0 | 0 | 0 | 0 | 0 | 0 | 0 | 0 | 0 | 0 | 0 |
| R1/LOA/Jockey | 0 | 0 | 0 | 0 | 0 | 0 | 0 | 0 | 0 | 0 | 0 | 0 | 0 | 0 | 0 | 0 | 0 | 0 | 0 | 0 | 0 | 0 | 0 | 0 | 0 |
| R2/R4/NeSL | 0 | 0 | 0 | 0 | 0 | 0 | 0 | 0 | 0 | 0 | 0 | 0 | 0 | 0 | 0 | 0 | 0 | 0 | 0 | 0 | 0 | 0 | 0 | 0 | 0 |
| RTE/Bov-B | 0 | 0 | 0 | 0 | 0 | 0 | 0 | 0 | 0 | 0 | 0 | 0 | 0 | 0 | 0 | 0 | 0 | 0 | 0 | 0 | 0 | 0 | 0 | 0 | 0 |
| L1/CIN4 | 0 | 0 | 0 | 0 | 0 | 0 | 0 | 0 | 0 | 0 | 0 | 0 | 0 | 0 | 0 | 0 | 0 | 0 | 0 | 0 | 0 | 0 | 0 | 0 | 0 |
| LTR elements | 315 | 363 | 285 | 341 | 212 | 689 | 505 | 174 | 211 | 208 | 260 | 413 | 321 | 232 | 369 | 235 | 412 | 450 | 606 | 393 | 558 | 382 | 266 | 692 | 548 |
| BEL/Pao | 0 | 0 | 0 | 0 | 0 | 0 | 0 | 0 | 0 | 0 | 0 | 0 | 0 | 0 | 0 | 0 | 0 | 0 | 0 | 0 | 0 | 0 | 0 | 0 | 0 |
| Ty1/Copia | 0 | 0 | 0 | 0 | 0 | 0 | 0 | 0 | 0 | 0 | 0 | 0 | 0 | 0 | 0 | 0 | 0 | 0 | 0 | 0 | 0 | 0 | 0 | 86 | 0 |
| Ty3/Gypsy | 26 | 25 | 40 | 30 | 18 | 36 | 45 | 11 | 15 | 14 | 10 | 16 | 25 | 11 | 20 | 9 | 30 | 26 | 32 | 29 | 32 | 21 | 24 | 281 | 30 |
| Retroviral | 0 | 0 | 0 | 0 | 0 | 0 | 0 | 0 | 0 | 0 | 0 | 0 | 0 | 0 | 0 | 0 | 0 | 0 | 0 | 0 | 0 | 0 | 0 | 0 | 0 |
| DNA transposons | 91 | 116 | 69 | 72 | 44 | 603 | 496 | 95 | 140 | 217 | 230 | 289 | 136 | 173 | 132 | 110 | 153 | 149 | 464 | 86 | 108 | 235 | 51 | 539 | 206 |
| hobo-Activator | 0 | 0 | 0 | 0 | 0 | 0 | 0 | 1 | 0 | 0 | 18 | 10 | 2 | 2 | 1 | 6 | 0 | 1 | 1 | 0 | 2 | 0 | 0 | 0 | 0 |
| Tc1-IS630-Pogo | 0 | 0 | 0 | 0 | 0 | 0 | 0 | 0 | 0 | 0 | 0 | 0 | 0 | 0 | 0 | 0 | 0 | 0 | 1 | 0 | 0 | 5 | 0 | 201 | 27 |
| En-Spm | 0 | 0 | 0 | 0 | 0 | 0 | 0 | 0 | 0 | 0 | 0 | 0 | 0 | 0 | 0 | 0 | 0 | 0 | 0 | 0 | 0 | 0 | 0 | 0 | 0 |
| MuDR-IS905 | 0 | 0 | 0 | 0 | 0 | 0 | 0 | 0 | 0 | 0 | 0 | 0 | 0 | 0 | 0 | 0 | 0 | 0 | 0 | 0 | 0 | 0 | 0 | 0 | 0 |
| PiggyBac | 0 | 0 | 0 | 0 | 0 | 0 | 0 | 0 | 0 | 0 | 0 | 0 | 0 | 0 | 0 | 0 | 0 | 0 | 0 | 0 | 0 | 0 | 0 | 0 | 0 |
| Tourist/Harbinger | 0 | 0 | 0 | 0 | 0 | 0 | 0 | 0 | 0 | 0 | 0 | 0 | 0 | 0 | 0 | 0 | 0 | 0 | 0 | 0 | 0 | 0 | 0 | 0 | 0 |
| Other | 0 | 0 | 0 | 0 | 0 | 0 | 0 | 0 | 0 | 0 | 0 | 0 | 0 | 0 | 0 | 0 | 0 | 0 | 0 | 0 | 0 | 0 | 0 | 0 | 0 |
| Rolling-circles | 0 | 0 | 0 | 0 | 0 | 0 | 0 | 0 | 0 | 0 | 0 | 0 | 0 | 0 | 0 | 0 | 0 | 0 | 0 | 0 | 0 | 0 | 0 | 0 | 0 |
| Unclassified | 133 | 103 | 108 | 135 | 90 | 467 | 425 | 41 | 95 | 42 | 104 | 59 | 71 | 83 | 60 | 38 | 154 | 112 | 364 | 178 | 226 | 186 | 101 | 221 | 44 |
| Small RNA | 0 | 0 | 0 | 0 | 0 | 0 | 0 | 0 | 0 | 0 | 0 | 0 | 0 | 0 | 0 | 0 | 0 | 0 | 0 | 0 | 0 | 0 | 0 | 0 | 0 |
| Satellites | 0 | 0 | 0 | 0 | 0 | 0 | 0 | 0 | 0 | 0 | 0 | 0 | 0 | 0 | 0 | 0 | 0 | 0 | 0 | 0 | 0 | 0 | 0 | 0 | 0 |
| Simple repeats | 2152 | 1094 | 905 | 914 | 964 | 4002 | 3398 | 599 | 1059 | 105 | 374 | 203 | 97 | 323 | 92 | 178 | 712 | 240 | 857 | 1091 | 2568 | 296 | 845 | 3941 | 5706 |
| Low complexity | 185 | 85 | 40 | 44 | 102 | 400 | 341 | 81 | 136 | 27 | 119 | 51 | 24 | 129 | 22 | 40 | 28 | 23 | 26 | 46 | 68 | 15 | 39 | 528 | 1403 |
